# The impact of bimanual reach training with augmented position sense feedback on post-stroke upper limb somatosensory and motor impairment

**DOI:** 10.1186/s12984-025-01764-z

**Published:** 2025-12-09

**Authors:** Beverley C. Larssen, Ronan Denyer, Mahta Khoshnam Tehrani, Anjana Rajendran, Carlo Menon, Lara Boyd

**Affiliations:** 1 Graduate Program in Rehabilitation Sciences, University of British Columbia, Vancouver, BC Canada; 2 Graduate Program in Neuroscience, University of British Columbia, Vancouver, BC Canada; 3 Institute of Neuroscience, Université catholique de Louvain, Brussels, 1200 Belgium; 4 Biomedical and Mobile Health Technology Laboratory, Department of Health Sciences and Technology, ETH Zurich, Lengghalde 5, Zurich, 8008 Switzerland; 5 Menrva Research Group, Schools of Engineering Science and Mechatronic Systems Engineering, Simon Fraser University, Metro Vancouver, BC Canada; 6 Department of Physical Therapy, University of British Columbia, Vancouver, BC V6T 1Z4 Canada

**Keywords:** Chronic stroke, Upper extremity, Position sense, Proprioception, Motor control, Augmented feedback

## Abstract

**Background:**

Impaired arm position sense is a common somatosensory impairment after stroke, which significantly impacts the performance of functional activities using the upper limb. However, few clinical interventions target loss of position sense after stroke. Our aim was to use interlimb force-coupling to augment position sense of the stroke-affected arm during a bilateral reaching task and investigate the impact of training with this feedback manipulation on measures of arm position matching ability and both bilateral and unilateral motor control.

**Methods:**

Twenty-four participants with a history of stroke were randomized (*N* = 12/group) to perform mirrored bimanual aiming movements with either interlimb force-coupling (Augmented PF) or uncoupled symmetrical reaches with only visual feedback about movement position. Participants completed 11 sessions (295 bimanual reaches/session) using a Kinarm End-Point robot. Performance on measures of arm position sense (Arm Position Matching, APM), motor impairment (Fugl-Meyer Upper Limb, FM), motor function (Wolf Motor Function Test, WMFT), unilateral reach accuracy and speed (Visually Guided Reaching, VGR), and bilateral reach symmetry were collected before and after training to characterize changes in upper limb somatosensory and motor control performance.

**Results:**

APM Task Scores improved for both groups. This improvement was specifically observed through reduced APM variability, but not accuracy. FM scores also improved for both groups. The group that did not practice with force-coupling between limbs improved on measures of bilateral movement symmetry on a mirrored reaching task and had faster VGR movement times in post-test.

**Conclusion:**

Symmetrical reach training with or without augmented PF led to reduced motor impairment and benefited upper limb position matching ability by reducing APM variability. Augmenting position sense during reaching did not provide additional benefits for position matching accuracy. Advantages for unilateral movement speed and bilateral reach symmetry measures in the group that practiced without interlimb coupling may reflect specificity of practice effects due to similarity between test and training conditions for this group.

**Supplementary Information:**

The online version contains supplementary material available at 10.1186/s12984-025-01764-z.

## Introduction

The ability to accurately detect the position of our limbs without vision is dependent on our proprioceptive senses. Intact proprioception is essential for accurate dexterous movement and recovery of this domain of somatosensation is considered a pre-requisite for upper limb motor recovery [1]. Many individuals with stroke experience upper limb proprioceptive impairments [2–4]. Impairments can be either unilateral or bilateral [4] and can be quantified in terms of position matching accuracy and variability [5, 6]. Rehabilitation of somatosensation after stroke is not a routine part of clinical care [7, 8] and there are few interventions that specifically target the restitution of position sense post-stroke [9–11]. This may represent an oversight in clinical practice, since well-functioning proprioception has been shown to be critical for learning [12, 13] and adapting motor skills [14, 15]. Further, proprioceptive loss or impairment due to disease processes that affect both the peripheral and central nervous system are associated with deficits in motor control [16–19].

Training with augmented feedback (i.e., feedback that supplements or augments existing feedback sources) is one technique that can help recalibrate position sense [20–22]. For example, somatosensory discrimination training protocols use the less-affected upper limb as a reference of correctness, cueing individuals to compare how the sensations of the stroke-affected arm feel relative to the less-affected arm [10, 21]. This approach has been shown to recalibrate and improve position sense after stroke [21]. Recalibration of position sense has been associated with improvement in general motor control in neurologically intact individuals, after stroke, and other clinical populations [6, 11, 12, 22]. Greater improvements in proprioceptive function are realized when the proprioceptive training includes an active movement component, rather than passive limb motion alone [6]. This is consistent with evidence from a hand illusion paradigm where a greater magnitude of position sense recalibration was observed when participants actively moved their hand compared to when they experienced movement passively [23]. Taken together, current evidence suggests targeting recalibration of position sense may be a helpful ingredient in training regimes that aim to improve general upper limb motor capacity after stroke.

Evidence from neurologically healthy adults shows that arm position sense can be accurately interpreted from one side of the body and be used to accurately mirror-match with the other limb [5]. Other evidence demonstrates that targeting recalibration of position sense, including methods that provide augmented feedback to enhance intrinsic feedback, can transfer to general motor control after stroke [6]. Building on this past work, we designed a training protocol for chronic stroke participants (>6 months post-stroke; [24]) using a bimanual robot. Participants practiced a series of symmetrical bimanual reaches with either augmented position sense feedback from the less-affected upper limb via force-coupling of the robot arms, or with only visual feedback of both robot handle positions (without force-coupling). Both groups of participants engaged in unassisted physical practice with their stroke-affected arm; however, the interlimb force-coupling experienced by the augmented position sense feedback group enabled their less-affected arm to experience simultaneous sensation of the same movement and served as a supplementary reference of correct position-based feedback during task performance. The robot also allowed for real-time measurement of movement quality and performance superior to standard clinical measurements [25].

The goal of this study was to assess if bimanual training with augmented position sense feedback resulted in improvements in measures of position sense, motor impairment, and motor control in the stroke-affected arm when compared to bimanual reach training without force-coupling. We hypothesized that augmenting position sense feedback of the stroke-affected arm by force-coupling reaching movements to the less-affected arm would lead to greater training benefits compared to the visual feedback control condition without force-coupling, where participants are likely to rely primarily on visual feedback of hand position to reach to targets.

## Methods

### Participants

Sample size estimates were informed from an a-priori power calculation based on pilot data where changes in the Wolf Motor Function Test (WMFT) were assessed as a clinical measure of upper limb motor control and motor function. Our pilot data showed a WMFT mean improvement (pre-post training) of 6.91 s for the stroke-affected upper limb with a standard deviation of 11.048 s. For α = 0.05 and 1-β = 0.80, our pilot data indicated that 18 participants were needed for one-tailed t-test. Due to the addition of a second group, we increased our sample size resulting in 12 participants per group.

Participants were considered eligible if they were between the age of 40–80, had a previous history of stroke more than six months prior to baseline testing, and were able to follow verbal instructions. The Montreal Cognitive Assessment [26] as well as the Frenchay Aphasia Screening Test [27] were employed to ensure participants could provide consent and understand task instructions in English. Exclusion criteria included: diagnosed with neurological or psychiatric conditions other than stroke, dementia, comprehensive aphasia, history of head trauma, history or substance abuse, failure to see targets, or inability to extend and maintain the stroke-affected arm/hand inside a target for 500 ms on a Kinarm End-Point robot (Kinarm, Kingston, Ontario).

### Randomization

After recruitment, participants were randomly allocated to either a group that received augmented position sense feedback via force-coupled reaching or a visual feedback only group (no force-coupling). A custom Matlab (MathWorks, Natick, MA) script was used to stratify participants based on sex, age, time since stroke (months), and upper limb motor impairment (quantified by upper limb Fugl-Meyer assessment score out of 66 [28]). Our target allocation ratio was 1:1 across groups and experimental conditions. A single member of the study team who was not involved in the delivery of the study assessments and intervention, completed the randomization and communicated training group allocation to the study team.

### Ethics statement

This study was approved by the University of British Columbia Ethics Committee (Clinical Research Ethics Board #H17-00545). All participants read and signed a consent form prior to any experimental protocols. All data collection was performed at the University of British Columbia Hospital.

### Study design and procedures

**Fig. 1:**
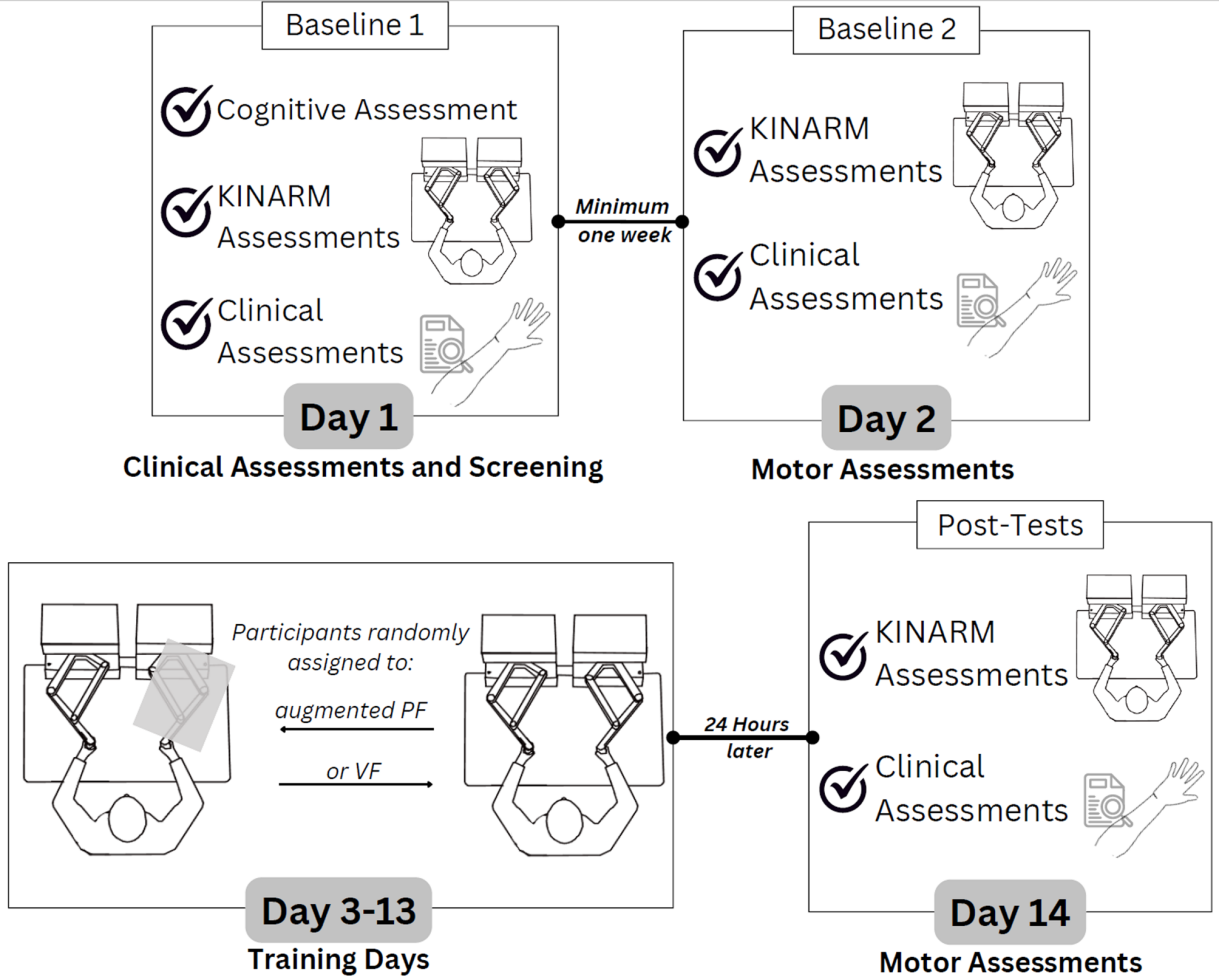
Diagram of study design. Baseline assessments were repeated at least one week apart before the 11-day training task where participants were randomly allocated to one of the two groups (Augmented Position Sense Feedback, PF; Visual Feedback, VF). A final post-test was completed 24 h after the last day of training

Experimental design and procedures are presented in Fig. 1. Baseline testing was repeated on two separate sessions at least one week apart to establish reliability and variability in outcome measures. Then, a minimum detectable change score was calculated and used to evaluate true change due to the reach training task (detailed below). After completion of the double baseline assessments, participants were randomly allocated to their respective groups and completed 11 training sessions (295 bimanual reaches/session) using a Kinarm End-Point Robot. All Kinarm data were collected using Dexterit-E version 3.6.2 and 3.8.0. Twenty-four hours after the final day of training, baseline assessments were repeated in a post-test.

### Assessments

During the baseline and 24-h post-test assessments, measures of arm position sense, bilateral movement symmetry, unilateral reach accuracy and speed, and clinical measures of upper limb motor impairment and function were quantified with the following assessments: Arm Position Matching (APM; position sense), uncoupled bimanual visually guided reaching (BVGR; custom robotic assessment of bilateral movement symmetry), Visually Guided Reaching (VGR; robotic assessment of unilateral reaching accuracy and speed), Wolf Motor Function Test (WMFT; clinical assessment of unilateral motor function), and the upper limb motor assessment from the Fugl-Meyer Assessment (FM; motor impairment). Clinical assessments (WMFT and FM) were administered by a physical therapist. Participants were also screened at the baseline timepoint for potential motor and/or position sense impairments in the less-affected upper limb using the VGR assessment and a custom assessment of unilateral position sense (One Arm Position Matching).

#### Arm position matching

APM is a standardized quantitative robotic assessment of upper limb passive position sense that is included in the suite of Kinarm standard tasks and has been validated against clinical measures of proprioception [5, 29]. Test-retest reliability for accuracy and variability parameters in healthy controls have been reported to be good (ICC = 0.88) and poor (ICC = 0.37) respectively [30]. This issue motivated the inclusion of a double-baseline assessment for quantifying minimum detectable change in our sample of individuals with stroke where we expected greater variability of scores due to the potential presence of impairment.

The assessment was completed in the horizontal plane in front of the participant. The robot passively moved the arm being assessed to one of 4 unseen target locations in the 2-dimensional workspace. Participants never had vision of either arm during the assessment. Once the robot stopped moving, the participant was asked to actively move their other arm controlling the other robot manipulandum to the mirror matched position in the workspace, and verbally indicate when they perceived that they had stopped in the accurate mirror-matched position. A total of 24 positions were sampled across 4 target locations during the task.

Different parameters were measured to characterize both general upper limb static position sense ability (APM task score) and specific features of static position sense (accuracy and variability).

i. APM Task Score is a composite score that represents overall task performance and served as a measure of general upper limb position sense ability. Task Scores are derived from parameter z-scores from discrete measures of accuracy and variability of position matching performance (described below). Detailed methods for this calculation are published elsewhere [31]. A value of zero is representative of best performance. Task Scores are always positive, with larger values being representative of worse performance. Scores exceeding 1.96 are considered impaired [31].
ii. Absolute Error XY was calculated as the mean absolute Euclidean distance error across all trials and served as a parameter measure of overall task accuracy. Higher error values are representative of poorer performance.
iii. Variability XY is the root-sum-square of the X and Y variability (mean standard deviation of error in the X and Y dimensions for all target locations) and was calculated to capture the variability in task errors.

#### One arm position matching

Due to the presence of somatosensory and motor impairments in the stroke-affected upper limb that could confound interpretation of less-affected arm position sense if assessed using the bilateral APM task, a custom assessment of unilateral position sense was developed for the Kinarm (One Arm Position Matching, OAP). Target locations were customized to match the corresponding 4 target locations assessed in the APM Kinarm standard task. During the OAP task, the participant held the robot handle with their less-affected hand. As with APM, participants never had vision of their upper limb or the robot handle during the task, but had intermittent visual feedback in the form of a white circle that aligned with the robot handle position that was projected on a reflective glass screen above the workspace. Participants first actively moved the robot handle and white circle to one of the 4 pre-defined peripheral target locations which was indicated by a green circle target (diameter: 2 cm). This position served as the start location for the trial. Once the start target location was reached, visual feedback of target location and robot handle position were extinguished. The robot then passively moved the assessed hand to the goal target location (movement duration: 1.5 s), where it paused for 0.5 s. During this time, participants were verbally cued to remember the goal target position. The robot then moved the assessed hand back to the start location (movement duration: 1.5 s). When the robot stopped, participants were asked to move their arm to the remembered goal target position and inform the examiner when they had replicated the remembered target location so the handle position could be recorded. The hand was then passively moved to a central location 10 cm away from all potential assessment targets. Next, a green target appeared at a new start location, the white circle representing the robot handle position reappeared on the screen, and the next trial began. The task comprised of 12 trials across the 4 possible goal target locations, in a randomized order (3 trials per target). Measures of position matching accuracy (OAP Absolute Error XY) and variability (OAP Variability XY) were calculated using the same calculation reported for APM Absolute Error XY and Variability XY described earlier. Both measures were then converted to a z-score based on the sample mean and standard deviation from a control data set of individuals without a history of stroke or upper limb somatosensory or motor impairments (Control group: *n* = 35, females = 24, right-handed = 33; median age = 39 years, range = 18–76 years; APM task scores < 1.96 in both limbs). Control group data for the OAP task are summarized in Supplementary Materials.

#### Bimanual visually guided reaching

In this task, two mirrored targets were projected on the screen, one for each hand. Participants were asked to move their arms together as fast and accurately as possible from one pair of green targets to the following pair of green targets. All movements for each hand started at a central green target and ended at one of 4 peripheral target locations 10 cm away (target diameter: 2.28 cm). Targets were presented in a random order. A total of 24 centre-out reaches were performed. Movements of the robot handles were not coupled, such that participants could move each hand independently, however they were encouraged to execute spatially symmetrical movements with the same timing and speed for both hands. Performance on this task was used to quantify spatiotemporal symmetry of bilateral reaches and was used as our motor control assessment of near transfer, given its similarity to the training tasks.

Symmetry in spatial performance was quantified with interlimb root mean square error (RMSE). For each movement trial, the spatial position of both robot handles (x, y coordinates across time, *t*) was sampled between the time of movement onset (when handle left central start circle, *t*_*i*_ = 1) and movement end (when hand reached peripheral target, *t*_*i*_ = *N*). The spatial position of the robot handle controlled by the more-affected arm relative to the other arm was calculated using the following equation:

$$\begin{gathered} {\text{Interlimb RMSE }} \hfill \\ = \sqrt {\frac{1}{N}\mathop \sum \limits_{{i = 1}}^{N} \,\left( \begin{gathered} \left( {x_{{right}} \left( {t_{i} } \right) - x_{{left}} \left( {t_{i} } \right)} \right)^{2} \, \hfill \\ + \,\left( {y_{{right}} \left( {t_{i} } \right) - y_{{left}} \left( {t_{i} } \right)} \right)^{2} \hfill \\ \end{gathered} \right)} \hfill \\ \end{gathered}$$

Mean interlimb RMSE averaged across all trials was calculated for both baseline (mean of baseline 1 and 2) and 24-h post-tests and used to characterize how well the participants were able to perform spatially symmetrical mirrored movements both pre-and post-intervention.

#### Visually guided reaching

Visually guided reaching (VGR) was included as a standardized assessment of unilateral upper limb motor control [32]. This task was selected to evaluate if any benefits conferred by bilateral reach training transfer to improvements in unilateral planar reaching in the stroke-affected upper limb. It was also used to test for potential impairments in the less-affected upper limb. Participants performed centre-out reaching movements from a central circular target to 4 peripheral targets located 10 cm away (target diameter: 1 cm). Participants were instructed to move as quickly and accurately as possible. A total of 20 centre-out reaches were performed. Different parameters were measured to characterize both general unilateral upper limb motor control (VGR Task Score) and specific features of movement quality related to spatial accuracy (path length ratio) and speed of movement (movement time).

i. VGR Task Score is a composite score that represents overall task performance and served as a measure of general upper limb motor control for unilateral reaching. VGR Task Scores are derived from parameter z-scores from discrete spatial and temporal variables measured during the VGR task (inclusive of spatial measures of accuracy, and temporal measures like reaction time and movement time). Detailed methods for this calculation are published elsewhere [31]. A value of zero is representative of best performance. Task Scores are always positive, with larger values being representative of worse performance. Scores exceeding 1.96 are considered impaired [31].
ii. Mean path length ratio served as a parameter measure of unilateral reaching accuracy. It is defined as the ratio of distance travelled by the hand between movement onset and movement termination and the shortest straight-line distance between the start and end target for each trial. Higher values are representative of poorer performance.
iii. Median movement time (MT) in seconds across all trials was calculated to evaluate unilateral planar reaching speed. MT was defined as the total time elapsed from movement onset to movement termination. MT assessed during this task has been shown to demonstrate excellent test-retest reliability [32].

#### Wolf motor function test

The WMFT is a reliable and valid test of post-stroke arm motor function and contains 15 timed movement tasks [33]. If no repetitions were completed within 120 s for a task, a zero score was assigned. Each task was characterized via calculation of rate (repetitions/60 second); higher rates show faster movements and better motor function [34]. WMFT rate was collected as a measure of far-transfer to identify if any benefits conferred by the training tasks generalize beyond planar reaching to potential improvements in motor function.

#### Fugl-Meyer assessment

The upper extremity portion of the FM (/66) was collected to characterize potential transfer to improvements in stroke-affected arm motor impairment, with higher scores reflecting less impairment [28]. The FM is a reliable measure of post-stroke motor impairment in the chronic stage of post-stroke recovery [35–37] and is the most commonly reported post-stroke upper limb motor impairment measure [38].

### Training task

Training took place on a Kinarm robot over 11 sessions. Each session was comprised of the same schedule. Every session started with participants performing an active range of motion assessment to quantify the farthest target that could be reached in the workspace. This distance was recorded and used to calibrate the difficulty of all training tasks such that the size of the reachable workspace was scaled relative to participant impairment. Workspace calibration ensured that participants were always training at the limit of their active range of motion. There were 5 possible task difficulty levels that corresponded to reach distances of approximately 10 cm (easiest) to 25 cm (hardest). An illustration of the workspace range of motion for each task difficulty level has been provided in the Supplementary Materials.

Following the range of motion assessment, participants performed a series of symmetrical/mirrored bimanual reaching tasks in the horizontal plane to stationary and moving visual targets (295 reaches per session; 3,245 total). Task descriptions, including details of the visual stimuli and instructions given to the participants are provided in Supplementary Materials. Both participant groups performed the same tasks in the same order. Groups differed with respect to how augmented feedback was provided during the training tasks. The Augmented PF group was encouraged to actively move both arms together, however the experimental task behaviour was dependent on the active movement of the stroke-affected arm. If the stroke-affected arm was not actively moving the robot handle, a brake was applied. This served as feedback to encourage participants to intentionally reach with their stroke-affected hand. The robot arm controlled by the less-affected arm was force-coupled to the robot arm controlled by the stroke-affected arm. Vision of the stroke-affected arm was completely occluded such that participants could only see movement of a single white circle representing their less-affected arm hand position. Participants in the Augmented PF group were encouraged to pay attention to the sensation of movement of the less-affected arm to inform the spatial accuracy of the stroke-affected arm. The visual feedback group (VF) did not experience force-coupling of the robot arms during the reaching intervention, such that each arm could move independently. Also, unlike the Augmented PF group, VF participants were able to see two white circles representing the actual spatial location of both hands for all movements.

For analysis of performance during the training days, the Mirror Extension Task was selected as an exemplar task (See Supplementary Materials for task description). Interlimb RMSE was calculated between the spatial position (x, y coordinates across time) of the robot hand controlled by stroke-affected limb relative less-affected limb across each trial. Interlimb RMSE was used to characterize how well the participants were able to perform spatially symmetrical mirrored movements under the feedback constraints imposed on the respective groups. This measure also served as a manipulation check that the force-coupling experienced by participants training with Augmented PF was indeed encouraging movements with greater spatiotemporal symmetry.

### Statistical analysis

Task Scores and z-scores for parameters from the APM and VGR Kinarm standard tasks were first analyzed using Dexterit-E Explorer analysis version 3.9.3. Task and z-scores for Kinarm standard tasks are calculated relative to a normative data set and account for age, sex, and handedness effects that were present in the normative distributions [31]. Methods for calculating standardized scores are detailed elsewhere [31, 39]. Parameters derived from performance on custom tasks (One Arm Position Matching, Bimanual VGR, Mirror Extension) were calculated using a custom Matlab script (MATLAB R2019b, MathWorks, Natick, MA).

Statistical analyses were carried out using SPSS (SPSS 27.0; IBM Corporation, Armonk, NY) and R studio [40–42]. Data were tested for normality with the Shapiro-Wilk test and heterogeneity of variance using Levene’s test with α = 0.001 [43]. When data did not meet assumptions of parametric tests, an appropriate non-parametric test was used.

Participant demographics (Age, Time Since Stroke) were compared between groups using two separate independent samples t-tests. Non-parametric Mann-Whitney U tests were used for non-parametric data.

To evaluate test-retest reliability of all baseline clinical (FM, WMFT rate) and Kinarm assessments (APM Task Score, APM AbsXY z-score, APM VarXY z-score, VGR Task Score, VGR path length ratio z-score, VGR MT z-score, Bimanual VGR interlimb RMSE) from Baseline 1 to Baseline 2, separate intra-class correlation coefficients (ICC) and their respective 95% confidence intervals were calculated using a two-way mixed effects model, single measurement protocol with absolute agreement [44]. ICC’s and the standard deviation (SD) of the mean difference between Baseline 1 and Baseline 2 measures were used to calculate a minimum detectable change (MDC) score for each outcome measure. MDC is defined as the smallest amount of change that is not due to inherent variation or noise in the measure itself, and has been used by others as the minimum threshold to detect change in an outcome measure that can be attributed to an interleaving intervention [45]. The following equation was used to calculate the 90% confidence interval of a reliable difference (MDC_90_) as reported by others who have assessed rehabilitation and motor learning outcome measures [45–48]: MDC_90_ = 1.65 X SD_Baseline_ X $\:\sqrt{2[1-ICC]}$ .

Linear mixed effects regression (MER) was used to evaluate changes in bilateral spatial symmetry (interlimb RMSE during the Mirror Extension task) across the 11 days of training. Group, training day, and their interaction were included as fixed effects in the model. Group (Augmented PF versus VF, reference level = VF) was included as a categorical between-subjects fixed effect and training days 1 to 11 were modeled as a continuous repeated measure fixed effect. Participant was included in the model as a random intercept. Model fit was evaluated using Akaike’s information criterion (AIC). The linear model and inclusion of Participant as a random effect produced the best fit (lowest AIC).

To evaluate the effectiveness of training, separate repeated measures ANOVA (rmANOVA) were used to evaluate differences in performance between baseline (average score of Baseline 1 and 2) and the 24 h post-test for clinical and Kinarm assessments. Group (Augmented PF versus VF) and Time (Baseline versus Post-test) were entered as between and repeated measures factors, respectively. Partial eta squared ($\:{\eta\:}_{p}^{2}$) effect sizes are reported for all tests and observed power (*1-β*) is reported for all non-significant effects. Significant interactions were followed up with post-hoc pairwise t-tests with Bonferroni correction applied. Post-hoc descriptive comparisons between Post-test minus Baseline change scores and the MDC_90_ score for each outcome measure are also reported. Exploratory post-hoc Spearman’s correlations were used to assess potential relationships between baseline sensory impairment (APM Task Score: stroke-affected arm; OAP VarXY and AbsXY parameters: less-affected arm) and Post-test minus Baseline change scores on the position matching task. Exploratory correlations were also used to assess relationships between baseline motor impairment (Fugl-Meyer score) and Post-test minus Baseline changes scores for unilateral motor outcomes (VGR Task Score, FM).

## Results

Twenty-six participants were enrolled between 2018 and 2022. Participant recruitment was paused between March 2020 and November 2020 owing to the COVID-19 pandemic. Two participants dropped out after the first session of baseline assessments because they were not interested in continuing in the study. A total of 24 individuals with stroke (*n* = 12 Augmented PF; *n* = 12 VF) completed the study protocol. There were no adverse events associated with the assessment or training protocols.

### Baseline assessments and participant demographics

Participant demographics are reported in Table 1. There were no group-level differences in mean age (*t*(22) = 0.41, *p* = 0.34), time since stroke (*t*(22) = 0.51, *p* = 0.31), or average baseline FM score between groups (*t*(22) = 0.33, *p* = .37). The median time to complete the training also did not differ between groups (*U* = 98.5, *p* = 0.12). Test-retest reliability statistics (ICC’s and 95% confidence intervals) and MDC_90_ score for all clinical and Kinarm assessments that were completed twice during baseline are also reported in Table 1. Criteria for categorizing ICC’s were based on previous guidelines [44]. Based on 95% CI’s for ICC estimates, test-retest reliability was excellent (greater than 0.9) for FM and WMFT Rate, and ranged from poor to moderate for all unilateral and bimanual VGR parameters. For measures of position sense, test-retest reliability ranged from good for APM Task Score to moderate and poor for APM Accuracy and Variability z-score ratings respectively. Impaired VGR performance was observed in the stroke-affected upper limb for 11 participants (VGR Task Scores >1.96; Augmented PF, *n* = 6; VF, *n* = 5). Impaired position sense was observed in the stroke-affected upper limb for 9 participants (APM Task Scores >1.96; Augmented PF, *n* = 4; VF, *n* = 5).

Motor control and position sense in the less-affected arm were also assessed using VGR and a custom assessment of unilateral position sense (OAP). Baseline scores and reliability statistics are also summarized in Table 1. Test-retest reliability of VGR Task Scores for the less-affected arm was poor. Impaired VGR performance based on average baseline scores was observed in the less-affected upper limb for 7 participants (VGR Task Scores > 1.96; Augmented PF, *n* = 4; VF, *n* = 3). Test-retest reliability of OAP accuracy and variability parameters were moderate and poor respectively. Z-scores calculated relative to a normative data set of control participants without a history of stroke or upper limb somatosensory or motor impairments, revealed that 13 participants with stroke had impaired performance in their less-affected arm for both OAP accuracy and variability measures (z-scores > 1.65; Augmented PF, *n* = 7; VF, *n* = 6).

**Table 1:** Participant demographics, training duration, baseline clinical assessment and Kinarm assessment scores

	Augmented PF *N* = 12	VF *N* = 12	ICC [95% CI]	MDC_90_
**Demographics**				
Sex (F/M)	4/8	3/9	-	-
Mean Age, years (SD)	66.69 (13.04)	64.88 (8.15)	-	-
Mean Time Since Stroke, months (SD)	100.56 (74.26)	86.78 (56.49)	-	-
Median training duration, days (IQR)	34 (32, 37)	38.5 (34, 49)	-	-
Stroke-affected arm (R/L)	5/7	5/7	-	-
**Clinical Assessments**				
Mean Fugl-Meyer, Total Score /66 (SD)	48.96 (13.77)	46.88 (17.04)	0.98 [0.96 − 0.99]	0.96 points
Mean WMFT Rate, repetitions/60 sec (SD)	32.52 (16.10)	31.65 (17.73)	0.95 [0.89 − 0.98]	2.73 reps/ 60 s
**Kinarm Assessments – stroke-affected arm**				
Mean APM Task Score (SD)	1.64 (0.94)	1.74 (1.20)	0.90 [0.78 − 0.96]	0.36
APM Accuracy				
Mean AbsXY Raw score, cm (SD)	6.41 (1.67)	7.13 (3.51)	-	-
Mean AbsXY Z-Score (SD)	0.47 (0.66)	0.46 (1.45)	0.79 [0.57 − 0.90]	1.20 Z
APM Variability				
Mean VarXY Raw score, cm (SD)	3.89 (1.41)	4.17 (1.81)	-	-
Mean VarXY Z-Score (SD)	0.34 (1.16)	0.50 (1.39)	0.65 [0.33 − 0.84]	2.07 Z
Mean VGR Task Score (SD)*	2.87 (1.54)	2.52 (1.63)	0.68 [0.63 − 0.85]	1.78
VGR Path Length Ratio				
Mean Raw score (SD)*	1.14 (0.07)	1.34 (0.69)	-	-
Mean Z-score (SD)*	0.77 (1.13)	0.73 (1.03)	0.62 [0.24 − 0.83]	1.29 Z
VGR Movement Time				
Mean MT, Raw score, seconds (SD)*	1.29 (0.25)	1.46 (0.92)	-	-
Mean MT Z-Score (SD)*	1.51 (1.21)	1.22 (1.01)	0.77 [0.53 − 0.90]	0.92 Z
**Kinarm Assessments – less-affected arm**				
OAP Accuracy				
Mean AbsXY Raw score, cm (SD)	3.65 (2.65)	2.96 (0.89)	-	-
Mean AbsXY Z-Score (SD)	3.56 (5.77)	2.06 (1.93)	0.88 [0.73 − 0.95]	-
OAP Variability				
Mean VarXY Raw score, cm (SD)	4.38 (3.47)	3.71 (1.39)	-	-
Mean VarXY Z-Score (SD)	4.16 (6.62)	2.89 (2.65)	0.67 [0.33 − 0.86]	-
Mean VGR Task Score (SD)	1.47 (0.76)	1.44 (0.91)	0.36 [-0.06 − 0.66]	-
**Bimanual VGR Assessment**				
Mean Interlimb RMSE (SD)*	2.00 (0.45)	2.28 (0.84)	0.75 [0.46 – 0.90]	0.57 cm

*Augmented PF*: Augmented position sense feedback,* ICC:* intra-class correlation coefficient, CI: confidence interval, MDC_90_: 90th percentile minimum detectable change score, *SD: * standard deviation,* IQR:* inter-quartile range,* WMFT *: Wolf Motor Function Test,* APM:* Arm Position Matching test,* VGR: * Visually guided reaching assessment,* MT:* movement time, OAP: one-arm position matching test,* RMSE:* root mean square error

*Mean values and ICC’s calculated based on reduced sample: stroke-affected arm VGR parameters (n = 11, Augmented PF; n = 9, VF), Bimanual VGR (n = 11, Augmented PF; n = 10, VF).

### Training task performance

i. Range of Motion Assessment

At the beginning of each training session, participants completed an active range of motion assessment to scale target distances for the day’s training tasks. Adapting training task difficulty based on maximum active reach distance and area achieved by the stroke-affected arm in the Kinarm workspace accommodated participants with a range of severe to mild upper limb motor impairments (Fugl-Meyer scores: 18–64). Maximum reach distance and area during the range of motion assessment did not change across practice days for all but 5 participants. Of the individuals who did experience change, 3 increased their maximum reach distance, which was maintained on subsequent sessions (average baseline Fugl-Meyer scores: 52, 47, 40, all in the VF group). Maximum reach distance fluctuated across days for the other 2 participants (average baseline Fugl-Meyer scores: 28, 51; both in the Augmented PF group). An illustration of the relationship between baseline motor impairment and maximum reach distance achieved by the stroke-affected arm in the active range of motion assessment is presented in the Supplementary Materials for descriptive purposes. The less-affected arm also completed the active range of motion assessment. Despite evidence of abnormal performance on the VGR task, this did not limit any participant’s ability to reach to all workspace targets with their less-affected arm.

ii. Mirror Extension interlimb RMSE

**Fig. 2:**
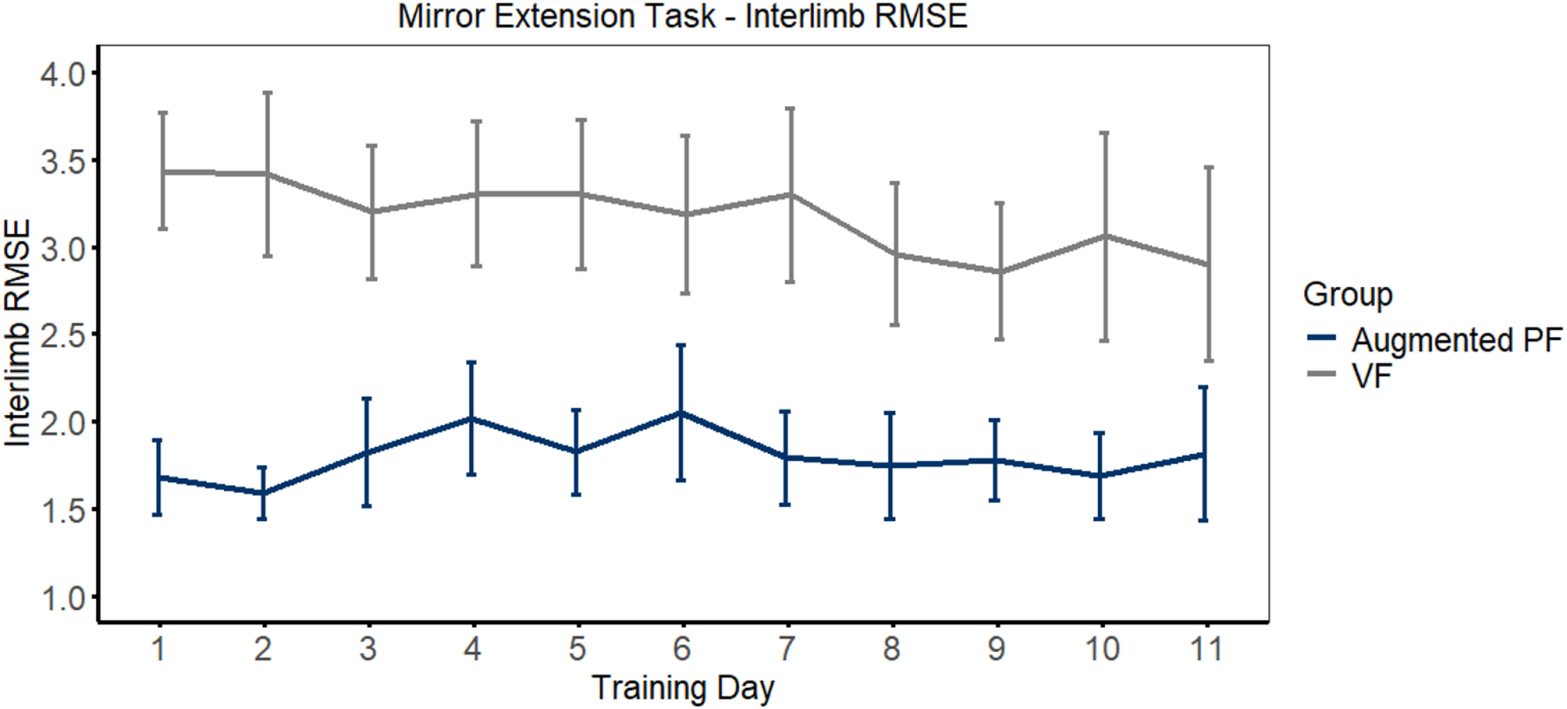
Mean interlimb root mean square error (RMSE) is plotted as a function of feedback group (Augmented PF vs. VF) and training day (1–11). Error bars represent standard error of the mean

The mirror extension task was selected as an exemplar task to illustrate changes in bilateral reach symmetry. Mean interlimb RMSE is plotted in Fig. 2. As expected, the Augmented PF group that reached with force-coupling between robot arms had lower interlimb RMSE than the VF group independent of training day (β = -1.64, CI [-2.64, − 0.65], *p* = .001). Interlimb RMSE decreased as function of training day (β = − 0.05, CI [-0.09, − 0.01], *p* = .01), however this was likely driven by the VF group as evidenced by the Group X training day interaction, which approached statistical significance (β = 0.05, CI [0, 0.11], *p* = .050). The full model output is presented in Table 2.

**Table 2:** Results of linear mixed effects regression analysis of interlimb root mean square error (RMSE) data during the mirror extension training task

	Mirror Extension Task: Interlimb RMSE		
Predictors	Estimates	95% CI	*p*
(Intercept)	3.46	2.78–4.15	**< 0.001**
Training Day	-0.05	-0.09 – -0.01	**0.010**
Feedback Group [Augmented PF vs VF]	-1.64	-2.64 – -0.65	**0.001**
Training Day × Feedback Group [Augmented PF vs VF]	0.05	-0.00–0.11	0.050
Random Effects			
σ^2^	0.45		
τ_00 Participant_	1.27		
ICC	0.74		
N _Participant_	23		
Observations	251		
Marginal R^2^ / Conditional R^2^	0.208 / 0.791		

Regression model equation: interlimb RMSE ~ 1 + Feedback group*Training Day + (1|Participant). lme4 notation used. Fixed effects: Feedback group (2 categories: Augmented PF versus VF), training data (continuous, 1–11). Random effect: Participant

### Effect of training on upper limb position sense, unilateral and bilateral motor control

#### Assessment of position sense

i. APM Task Score

**Fig. 3:**
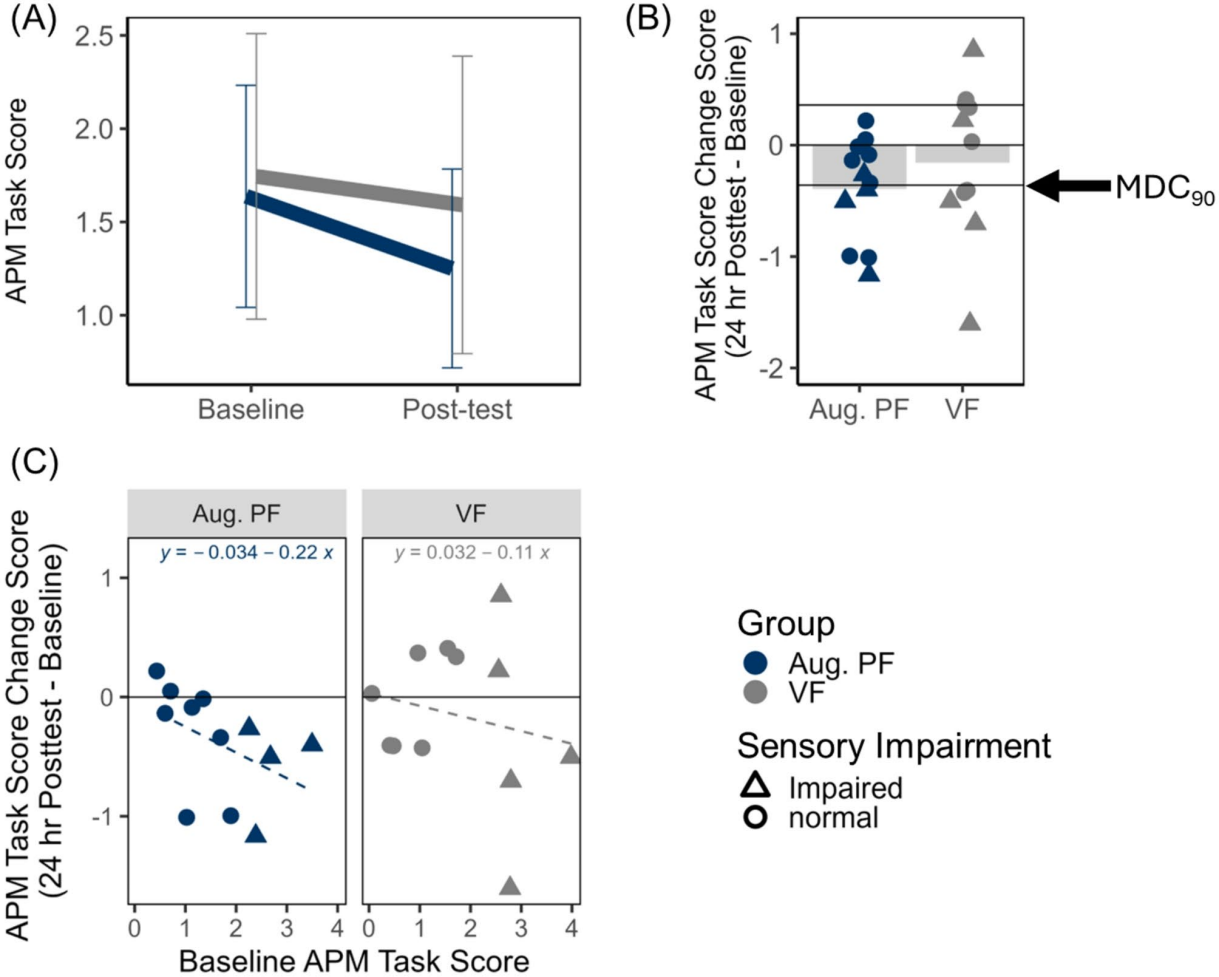
Arm position matching (APM) Task Score.** A** Mean task score plotted as a function of feedback group (Aug. PF = Augmented position sense feedback; VF = visual feedback only) and time (Baseline versus 24-h post-test). Error bars represent 95% confidence intervals.** B** Mean change in in Task Score (24-h post-test – Baseline) plotted relative to the MDC_90_ threshold (0.36). Negative change scores represent improvement in position sense. Group means are represented by shaded bars. Individual subject scores are represented by individual points on the plot, where triangles represent individuals whose baseline Task Score was impaired relative to normative values (> 1.96).** C** Within-group correlations between Baseline APM Task Score and change score

APM Task Score data are visualized in Fig. 3. Overall position sense, measured by APM Task Score, improved for both groups from baseline (Mean = 1.69, SD = 1.06) to post-test (Mean = 1.42, SD = 1.06) as evidenced by the main effect of Time, *F*(1, 22) = 5.50, *p* = 0.03, $\:{\eta\:}_{p}^{2}$ = 0.20. This did not vary with Group (*F* (1, 22) = 1.04, *p *= 0.32, $\:{\eta\:}_{p}^{2}$ = 0.05, 1-β = 0.16) nor was the Group main effect statistically significant (*F* (1, 22) = 0.28, *p *= 0.60, $\:{\eta\:}_{p}^{2}$ = 0.01, 1-β = 0.08). Changes in Task Score representing improvement (reduced score) that exceeded the MDC_90_ were observed in 5/12 participants in the Augmented PF group and 5/12 in the VF group (see Fig. 3, panel B). From our post-hoc exploratory correlations, we observed that the amount of improvement in APM Task Score was associated with severity of average baseline APM performance (represented by average APM Task Score from Baseline days 1 and 2) for the Augmented PF group (*r*_*s*_ = − 0.63, *p* = .03), but not the VF group (*r*_*s*_ = −0.30, *p* = 0.15). Participants in the Augmented PF group with greater position sense impairment at baseline experienced the greatest improvement in position sense score, however after correction for multiple comparisons, this effect was no longer statistically significant (Bonferroni-adjusted *p* = 0.09).

Since the intervention for the Augmented PF group also relies on reliable position sense from the less-affected arm, we tested if position sense of the less-affected upper limb (OAP AbsXY and VarXY parameters assessed at baseline) was associated with the change in APM Task Score. No statistically significant relationships were observed between measures for the Augmented PF group (OAP VarXY: *r*_*s*_ = − 0.36, *p* = 0.25; OAP AbsXY: *r*_*s*_ = − 0.25, *p* = 0.43) or the VF group (OAP VarXY: *r*_*s*_ = 0.11, *p* = 0.73; OAP AbsXY: *r*_*s*_ = 0.15, *p* = 0.65 ).

ii. APM Accuracy

**Fig. 4:**
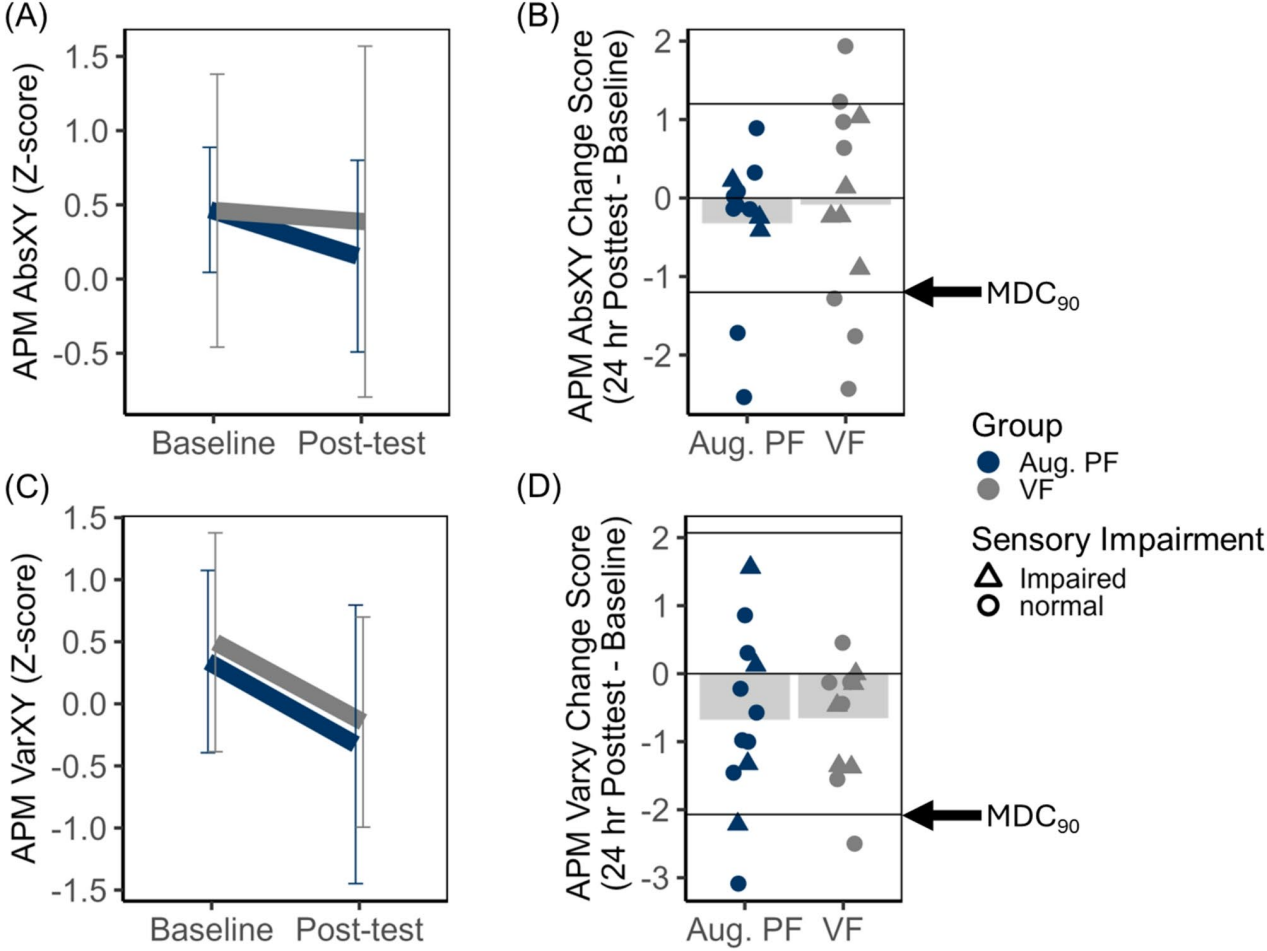
APM accuracy (AbsXY z-scores) and APM variability (VarXY z-scores).** A** Mean AbsXY z-score plotted as a function of feedback group (Aug. PF = Augmented position sense feedback; VF = visual feedback only) and time (Baseline versus 24-h Post-test). Error bars represent 95% confidence intervals.** B** Mean change in AbsXY z-score (24-h Post-test – Baseline) plotted relative to the MDC_90_ threshold (1.20).** C** Mean VarXY z-score plotted as a function of feedback group and time (Baseline versus 24-h Post-test). Error bars represent 95% confidence intervals.** D** Mean change in VarXY z-score (24-h Post-test – Baseline) plotted relative to the MDC_90_ threshold (2.07). For panels B and D, negative change scores represent improvement in position sense. Group means are represented by shaded bars. Individual subject scores are represented by individual points on the plot, where triangles represent individuals whose baseline APM Task Score was impaired relative to normative values (> 1.96)

APM accuracy measured using Absolute XY z-score data are visualized in Fig. 4 (panels A and B). APM accuracy did not improve from baseline to post-test (Time, *F* (1, 22) = 0.69, *p* = 0.42, $\:{\eta\:}_{p}^{2}$ = 0.03, 1-β = 0.13) and did not differ by group (Group, *F* (1, 22) = 0.54, *p *= 0.82, $\:{\eta\:}_{p}^{2}$ = 0.002, 1-β = 0.56; Group X Time, *F* (1, 22) = 0.26, *p *= 0.61, $\:{\eta\:}_{p}^{2}$ = 0.01, 1-β = 0.08). Improvements in APM accuracy that exceeded the MDC_90_ were observed in 2/12 participants in the Augmented PF group and 3/12 in the VF group (see Fig. 4, panel B).

iii. APM Variability

APM variability or precision in position matching performance, measured using Variability XY z-score data are visualized in Fig. 4 (panels C and D). APM variability improved for both groups from baseline (Mean = 0.42, SD = 1.25) to post-test (Mean = − 0.24, SD = 1.53) as evidenced by the statistically significant main effect of Time (*F* (1, 22) = 8.49, *p* = 0.008, $\:{\eta\:}_{p}^{2}$ = 0.28). This did not vary with Group (*F* (1, 22) = 0.003, *p *= 0.96, $\:{\eta\:}_{p}^{2}$ < 0.001, 1-β = 0.05) nor was the Group main effect statistically significant (*F* (1, 22) = 0.05, *p *= 0.82, $\:{\eta\:}_{p}^{2}$ = 0.002, 1-β = 0.06). Changes in APM variability that exceeded the MDC_90_ were observed in 2 participants in the Augmented PF group and 1 VF participant (see Fig. 4, panel D).

#### Transfer to assessment of unilateral reaching

Four participants had to be excluded from the analysis of VGR task parameters (3 did not have adequate active voluntary movement in their stroke-affected arm to complete the task at either timepoint; 1 participant had missing data for this task at post-test).

i. Unilateral VGR Task Score

**Fig. 5:**
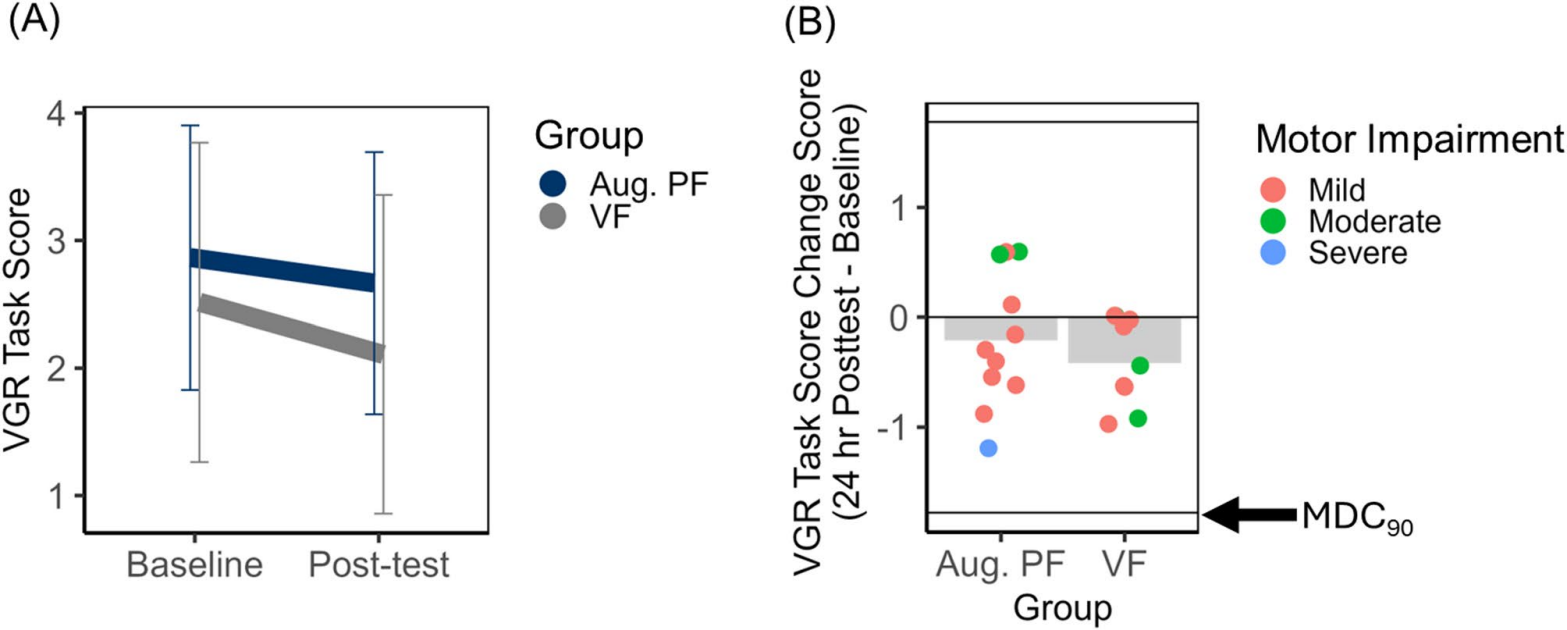
Unilateral motor control. Visually guided reaching (VGR) Task Score.** A** Mean VGR Task Score plotted as a function of feedback group (Aug. PF = Augmented position sense feedback; VF = visual feedback only) and time (Baseline versus 24-h Post-test). Error bars represent 95% confidence intervals. **B** Mean change in VGR Task Score (24-h Post-test – Baseline) plotted relative to the MDC_90_ threshold (1.78). Negative change scores represent improvement in overall task performance. Group means are represented by shaded bars. Individuals subject scores are represented by individual points on the plot, disaggregated by established cut-offs for severe, moderate, and mild upper limb motor impairment defined by Fugl-Meyer score [49]

VGR Task Score data are visualized in Fig. 5. VGR Task Score decreased from baseline to post-test for both groups as evidenced by the main effect of Time (*F* (1, 18) = 6.55, *p* = 0.02, $\:{\eta\:}_{p}^{2}$ = 0.27). The main effects of Group (*F* (1, 18) = 0.42, *p* = 0.52, $\:{\eta\:}_{p}^{2}$ = 0.02, 1-β = 0.09) and Group X Time interaction (*F* (1, 18) = 0.76, *p* = 0.40, $\:{\eta\:}_{p}^{2}$ = 0.04, 1-β = 0.13) were not statistically significant. Despite evidence of a statistically significant change in VGR Task Score from baseline to post-test, no participants exceeded the MDC_90_ for this outcome measure (see Fig. 5, panel B). From post-hoc exploratory correlations we did not observe evidence of a statistically significant relationship between the amount of improvement in VGR task score and baseline upper limb motor impairment (average Fugl-Meyer score from Baseline days 1 and 2) in either the Augmented PF (*r*_*s*_ = −0.18, *p* = 0.44) or the VF group (*r*_*s*_ = −0.20, *p* = 0.61). This suggests that improvements in general unilateral reaching motor control were independent of overall upper limb motor impairment severity.

ii. Unilateral VGR Path Length Ratio Z-Score

**Fig. 6:**
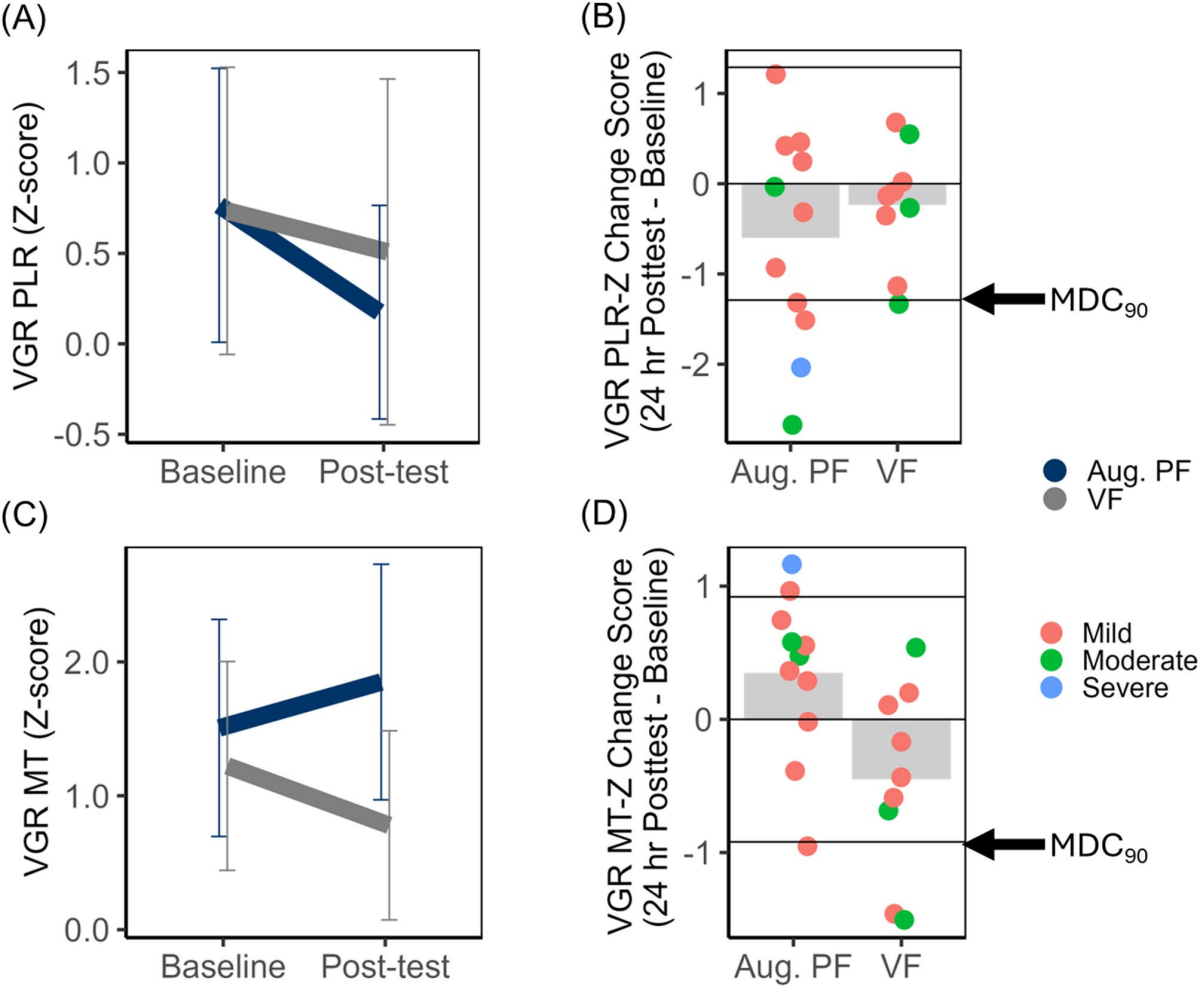
Unilateral motor control. Visually guided reaching (VGR) path length ratio (PLR z-scores) and movement time (MT z-scores).** A** Mean VGR PLR z-score plotted as a function of feedback group (Aug. PF = Augmented position sense feedback, VF = visual feedback only) and time (Baseline versus 24-h Post-test). Error bars represent 95% confidence intervals.** B** Mean change in in PLR z-score (24-h Post-test – Baseline) plotted relative to the MDC_90_ threshold (1.29). **C** Mean VGR MT z-score plotted as a function of feedback group and time (Baseline versus 24-h Post-test. Error bars represent 95% confidence intervals. **D** Mean change in MT z-score (24 hour Post-test – Baseline) plotted relative to the MDC_90_ threshold (0.92). Negative change scores represent improvement for each motor control parameter. Group means are represented by shaded bars. Individuals subject scores are represented by individual points on the plot, disaggregated by established cut-offs for severe, moderate, and mild upper limb motor impairment defined by Fugl-Meyer score [49].

With respect to spatial accuracy of trajectories, path length ratio z-scores appeared to decrease for both groups, however the main effect of Time only approached statistical significance (*F* (1, 18) = 3.32, *p* = 0.09, $\:{\eta\:}_{p}^{2}$ = 0.16, 1-β = 0.41). Neither the main effect of Group (*F*(1, 18) = 0.13, *p* = 0.73, $\:{\eta\:}_{p}^{2}$ = 0.01, 1-β = 0.06) or the Group X Time interaction (*F* (1, 18) = 0.66, *p* = 0.43, $\:{\eta\:}_{p}^{2}$ = 0.04, 1-β = 0.12) were statistically significant. Improvements in path length ratio z-score that exceeded the MDC_90_ were observed in 4/12 participants in the Augmented PF group and 1/12 in the VF group (see Fig. 6, panel B).

iii. Unilateral VGR Movement Time (MT) Z-Score

Movement time z-score decreased from baseline to post-test for only the VF group as evidenced by the Group X Time interaction, *F* (1, 18) = 7.22, *p* = 0.02, $\:{\eta\:}_{p}^{2}$ = 0.29. The main effects of Group (*F* (1, 18) = 1.91, *p* = 0.18, $\:{\eta\:}_{p}^{2}$ = 0.10, 1-β = 0.26) and Time (*F* (1, 18) = 0.12, *p* = 0.74, $\:{\eta\:}_{p}^{2}$ = 0.01, 1-β = 0.06) were not statistically significant. MT z-score data are presented in Fig. 6. Despite the lower MT at post-test for the VF group (confirmed with post-hoc testing, *p* < 0.05), only 2 VF and 1 Augmented PF participants demonstrated a change in MT z-score that exceeded the MDC_90_ threshold (Fig. 6, panel D).

#### Transfer to assessment of bilateral reaching: Bimanual VGR Interlimb RMSE

**Fig. 7:**
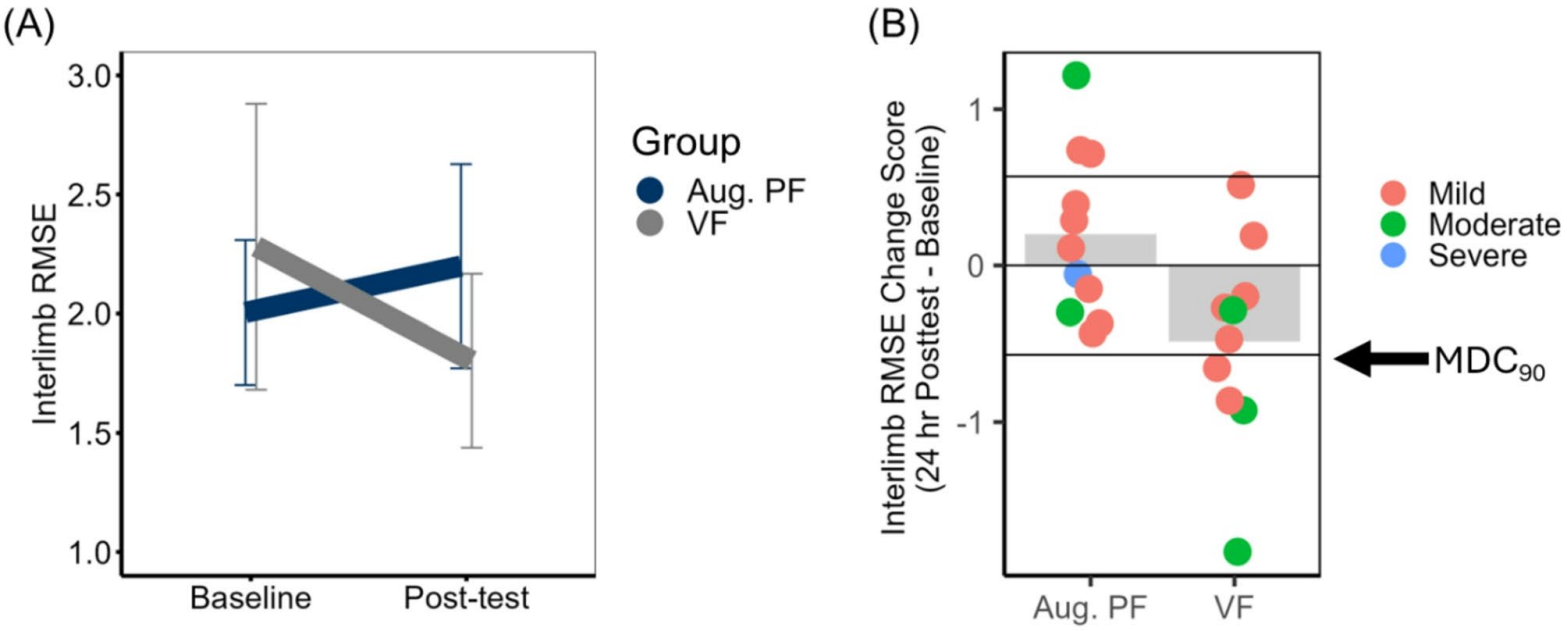
Bilateral motor control. Bimanual visually guided reaching (BVGR) interlimb root mean square error (RMSE).** A** Mean interlimb RMSE plotted as a function of feedback group (Aug. PF = Augmented position sense feedback; VF = visual feedback only) and time (Baseline versus 24-h Post-test). Error bars represent 95% confidence intervals.** B** Mean change in interlimb RMSE (24 hour Post-test – Baseline) plotted relative to the MDC_90_ threshold (0.57 cm). Negative change scores represent improvement in spatiotemporal movement symmetry. Group means are represented by shaded bars. Individuals subject scores are represented by individual points on the plot, disaggregated by established cut-offs for severe, moderate, and mild upper limb motor impairment defined by Fugl-Meyer score [49]

Three participants had to be excluded from the analysis of interlimb RMSE during the Bimanual VGR task (all did not have adequate voluntary movement in their stroke-affected arm to complete this task at either timepoint and were the same individuals that could not complete the unilateral VGR task). Interlimb RMSE decreased as a result of the intervention for only the VF group, as evidenced by the Group X Time interaction, *F* (1, 19) = 6.83, *p* = 0.02, $\:{\eta\:}_{p}^{2}$ = 0.26 and confirmed with post-hoc pairwise t-tests (*p* = 0.04). As presented in Fig. 7 (panel B), only the VF group had participants that reduced their RMSE such that it exceeded the MDC_90_ threshold (*n* = 4). The main effects of Group (*F* (1, 19) = 0.06, *p* = 0.81, $\:{\eta\:}_{p}^{2}$ = 0.003, 1-β = 0.06) and Time (*F* (1, 19) = 1.22, *p* = 0.28, $\:{\eta\:}_{p}^{2}$ = 0.06, 1-β = 0.18) were not statistically significant.

#### Transfer to clinical assessments of unilateral upper limb motor impairment and function

i. Fugl-Meyer

**Fig. 8:**
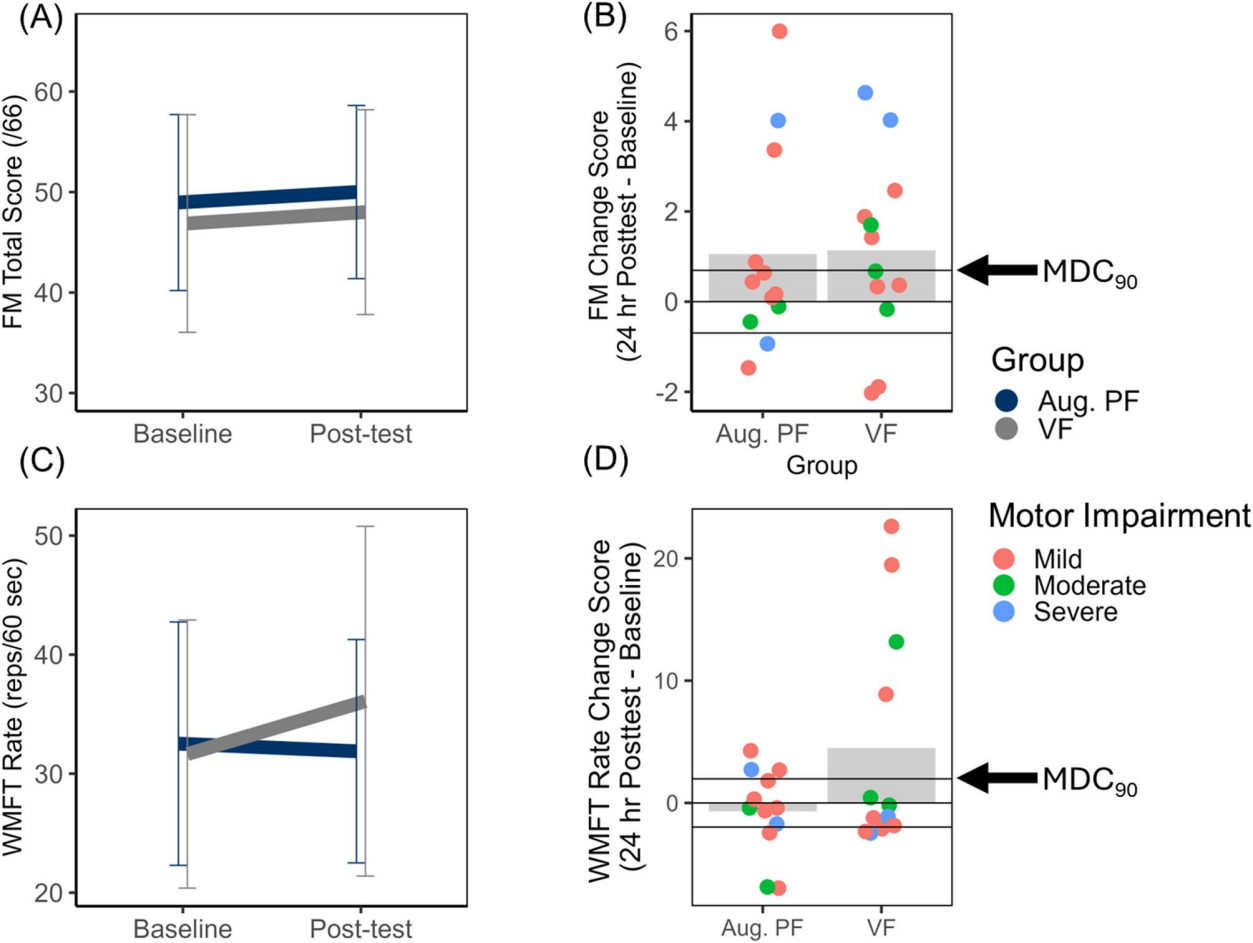
Clinical assessments of unilateral motor impairment and motor function.** A** Mean Fugl-Meyer (FM) total score (/66) plotted as a function of feedback group (Aug. PF = Augmented position sense feedback; VF = visual feedback only) and time (Baseline versus 24-h post-test). Error bars represent 95% confidence intervals.** B** Mean change in FM (24-h Post-test – Baseline) plotted relative to the MDC_90_ threshold (0.97 points).** C** Mean Wolf motor function test (WMFT) rate (repetitions/minute) plotted as a function of group and time (Baseline versus 24-h post-test). Error bars represent 95% confidence intervals.** D** Mean change in WMFT rate (24-h Post-test – Baseline) plotted relative to the MDC_90_ threshold (2.73 reps/min). For panels B and D, positive change scores represent improvement. Group means are represented by shaded bars. Individuals subject scores are represented by individual points on the plot, disaggregated by established cut-offs for severe, moderate, and mild upper limb motor impairments defined by FM score [49]

Upper limb motor impairment improved for both groups from baseline (Mean = 47.92 points, SD = 15.19) to post-test (Mean = 49.00 points, SD = 15.56) as evidenced by a statistically significant main effect of Time *F*(1, 22) = 6.15, *p* = 0.02, $\:{\eta\:}_{p}^{2}$ = 0.22. This did not vary with Group (*F* (1, 22) = 0.01, *p *= 0.93, $\:{\eta\:}_{p}^{2}$ < 0.001, 1-β = 0.05) nor was the Group main effect statistically significant (*F* (1, 22) = 0.11, *p*= 0.74, $\:{\eta\:}_{p}^{2}$ = 0.01, 1-β = 0.06). Improvements in FM total score that exceeded the MDC_90_ were observed in 4 participants in the Augmented PF group and 6 VF participants (see Fig. 8, panel B). No correlations between baseline impairment and FM change score were observed in either group (*p*s > 0.27). Additional exploratory post-hoc omnibus Spearman’s correlations revealed that change in Fugl-Meyer (ΔFM = FM_post−test_ – FM_baseline_) was also not associated with change in specific element of unilateral motor control (accuracy or speed) measured with the VGR Task (ΔFM vs. ΔVGR task score, *r*_*s*_ = 0.24, *p* = 0.31; ΔFM vs. ΔVGR PLR z-score, *r*_*s*_ = 0.39, *p* = 0.09; ΔFM vs. ΔVGR MT z-score, *r*_*s*_ = − 0.07, *p* = 0.78).

ii. WMFT rate

Performance on the WMFT did not improve for either groups evidenced by the absence of a statistically significant main effect of Time (*F* (1, 22) = 1.79, *p* = .19, $\:{\eta\:}_{p}^{2}$ = 0.08, 1-β = 0.25). The Group X Time interaction trended toward statistical significance (*F* (1, 22) = 3.20, *p *= 0.09, $\:{\eta\:}_{p}^{2}$ = 0.13, 1-β = 0.40), which was driven by the difference in post-test. The Group main effect also was not statistically significant (*F* (1, 22) = 0.05, *p *= 0.82, $\:{\eta\:}_{p}^{2}$ = 0.002, 1-β = 0.06). Improvements in WMFT rate that exceeded the MDC_90_ were observed in 3 participants in the Augmented PF group and 4 VF participants (see Fig. 8, panel D).

## Discussion

Our method of augmenting position sense feedback through real-time coupling of movements between two robot arms successfully improved the spatial symmetry of reaches compared to reaching without force-coupling between limbs. This method provided participants in the Augmented PF group with an alternative and more reliable source of position sense feedback from the less-affected upper limb, however, any potential benefit from reaching with this form of feedback was not different than performing uncoupled symmetrical reaches. This work demonstrates that multi-day practice of symmetrical bilateral reach training transfers to improvements in arm position sense, motor impairment and measures of motor control in the stroke-affected arm, independent of the type of augmented feedback provided. While this work is promising, improvements attained that exceeded the MDC_90_ criterion for each measure were less than 50% of the sample in both groups.

### Effects on position sense impairment

All participants were in the chronic stage of post-stroke recovery, so presumably position sense impairments were stable, yet after 11 training sessions, a measure of overall position sense (APM Task Score) improved for both groups. The amount of improvement was associated with severity of baseline impairment. Six of the 10 individuals in this sample whose position sense improved more than the MDC_90_ threshold had what would be considered impaired position sense at baseline (Task Score >1.96). Carey and colleagues [21] demonstrated that individuals with clinical diagnosis of position sense impairment can improve wrist position sense after engaging in upper limb tactile and proprioceptive discrimination training. Taken together these data suggest that position sense impairment may need to be present in order to realize any improvement in measures of position sense. Surprisingly, accuracy was not the primary source of position sense improvement, rather participants were able to reduce the variability of their performance on a position matching task. Across the whole sample, 18 participants were able to reduce the variability of their position matching error, yet only 3 of these individuals exceeded the MDC_90_. Given that the sample includes those with impairments, and position sense data is more variable when impairment is present after stroke [39, 50], the MDC_90_ for all measures of position sense is a conservative estimate of a threshold for true change when SDs are elevated. Similar effects of measurement variability and MDC have been observed in other outcome measures as well [45].

A surprising result from the current study is that force-coupling the less-affected hand position to the stroke-affected hand position during reaching behaviours did not provide a benefit compared to standard non-force-coupled bilateral reaches. We hypothesized that force-coupling would provide an added benefit over bilateral reaches by increasing the specificity of the proprioceptive input provided to the stroke-affected hand from the less-affected hand, with positive downstream consequences for motor control. Instead, comparable positive effects on outcome measures were observed for both feedback conditions. The lack of an additive effect of force-coupling may have been due to an inherent tendency for humans to spatiotemporally couple bimanual movements regardless as to whether force-coupling is applied. Previous research has shown that when participants are asked to simultaneously draw two different shapes with both hands, they tend to spontaneously spatially couple their movements, creating similar patterns between hands [51–54]. Bimanual reaches also tend to be strongly temporally coupled. For example, when reaches of different distances are performed, movement time tends to be similar between hands, which is facilitated by reducing the speed of the hand reaching the shorter distance [55–57]. The tendency for spatiotemporal coupling between hands seems to be relatively hard-wired within the motor system, as overcoming this constraint takes extensive training [58] and/or the deployment of perceptual tricks that change the cognitive representation of the task goals [59]. Thinking in these terms about the current study, the non-force-coupled condition may have generated similar outcomes to the force-coupling condition because of a natural tendency to spatiotemporally couple bimanual movements. This would mean that the less-affected hand was typically coupled with the stroke-affected hand, even in the absence of a correcting external forcefield. In other words, while our force-coupled intervention attempted to enrich the proprioceptive input available to the stroke-affected hand, greater spatiotemporal coupling may simply be a natural feature of bimanual reaching without any imposed external force-coupling. The interlimb RMSE data for the mirror extension task demonstrates that force-coupled bimanual reaches were more spatiotemporally coupled (i.e., lower RMSE) than non-force-coupled reaches. However, spatiotemporal coupling in the non-force-coupled condition remained relatively high. The average interlimb root mean squared error was ~ 3 cm in the non-force-coupled condition, compared to an average of ~ 1.75 cm in the force-coupled condition (Fig. 2).

Whether participants experienced force-coupling or not, the capacity to spatially couple bimanual movements was probably central to task success and accounts for the position sense improvement observed. This is particularly relevant in a stroke population since the corpus callosum, an area of the brain frequently damaged by stroke, is known to be the neural locus of spatial coupling of bimanual movements. Specifically, experiments with callosotomy patients demonstrate that surgically severing the corpus callosum leads to a deficit in spatial coupling of bimanual movements. This deficit only arises after the posterior portion of the corpus callosum, which connects the occipital and parietal lobes, is severed [60, 61]. A central feature of the data collected in the current study is that there was a high degree of heterogeneity in response to both force-coupled and non-force-coupled interventions. Given that stroke typically has highly heterogeneous effects in terms of lesion location and size, it may be the case that participants who showed less response to training had greater damage to the callosal tracts which seem to be critical for spatial coupling of bimanual movements. Future studies could investigate this by indexing callosal tract white matter structure and/or function using structural magnetic resonance imaging (MRI) and transcranial magnetic stimulation (TMS) techniques. Diffusion tensor imaging may be useful since it can index structural integrity of white matter in different subsections of the callosum [62, 63]. Dual coil TMS may also be useful as it can be used to measure transcallosal inhibition between primary motor [64, 65], premotor [66, 67], and non-motor regions including the parietal cortex [68]. If an absence of transcallosal inhibition as assessed by TMS or decreased structural integrity shown by MRI was associated with a lack of response to our bimanual intervention, this would provide strong evidence that the corpus callosum is a critical brain structure for this class of intervention. Such evidence could provide a biomarker for response to help clinicians make better informed decisions on specific indications for selecting the type of reach training intervention that will help improve upper limb somatosensory and motor recovery after stroke.

### Differential effects for measures of stroke-affected arm motor impairment versus motor function

Improved FM score was observed in 17 participants spanning all impairment severities, with 10 exceeding the MDC_90_ criterion. Repetitive reach practice led to improvement in upper extremity control in both the proximal and distal items of the scale. The task required repetitive practice of movements requiring voluntary control of the shoulder and elbow, which transferred to improvements on proximal items of the FM scale. This specific practice over and above usual rehabilitation activities was enough to facilitate some improvement for these participants, which is promising given a common critique that participants greater than 6 months post-stroke are often at a plateau and may not experience further recovery of motor impairment assessed with the FM [69]. There is mixed evidence about the efficacy of bilateral upper limb training for improvements in motor function [70–72]. In a recent systematic review and meta-analysis, Gnanaprakasam and colleagues concluded that measures of impairment improve after task-based bilateral movement training, however measures of activity with a speed component like the WMFT may not [72]. While this conclusion aligns with our results, it is not clear what mechanism is contributing to positive transfer to a measure of motor impairment for both feedback groups, but not motor function. Unilateral VGR Task Scores improved for both groups as well, however given that magnitude of Task Score change did not exceed the MDC_90_ for any participants in this sample, we are cautious about over-interpreting this finding. Change in motor impairment assessed with the FM was also not associated with change in measures of unilateral reaching accuracy (VGR path length ratio z-score) or speed (VGR movement time z-score), suggesting that neither motor control domain was contributing to the observed reduction in motor impairment. The absence of a relationship is perhaps not surprising given that the FM assessment does not have a speed constraint and is not sensitive to subtle differences in movement quality that can be captured by movement kinematics. Since all participants completed the same number of active movement repetitions with their stroke-affected arm, simply completing many repetitions of active movement with the stroke-affected arm, regardless of changes in movement speed or accuracy, may have been sufficient to strengthen the proximal muscle groups required to achieve FM test positions.

The VF group also notably demonstrated an advantage in the post-test on assessments of task-specific unilateral movement speed (VGR movement time) and bilateral movement symmetry, but not on a timed clinical assessment of motor function. Consistent with principles of practice specificity [73, 74], and evidence from the task-specific training literature [75, 76], we observed that when there was greater similarity between the training and assessment tasks, assessment scores improved. For example, the VF reaching condition without force-coupling experienced on the training days is most similar to both the unilateral and bimanual VGR reaching assessment. The VF training, unilateral VGR, and bimanual VGR assessments are all planar reaching tasks where the limbs are controlled independently and have identical visual feedback conditions. The WMFT assesses movement speed across a greater diversity of upper limb tasks requiring the control of more joints and degrees of freedom that were not specifically trained by reaching with the robot handle, which is likely why we did not observe transfer to this clinical assessment.

An alternative explanation for the VF group’s advantage on the unilateral and bimanual VGR tasks may be related to a partial guidance effects experienced by the Augmented PF group. In two of the training tasks to stationary targets (“Mirror Extension” and “Mirror Flexion”, see Supplementary Materials for task parameter descriptions), the Augmented PF group experienced additional guidance from force channels. Force channels and other partial guidance approaches require active movement, but restrict opportunities to detect and actively correct movement errors that may be important for motor learning and adaptation of reaching [77, 78]. The guidance hypothesis suggests that there is a detriment to learning when augmented feedback guides the learner toward correct performance [79, 80]. When learning an asynchronous bimanual motor control task, partial guidance of one limb through a desired movement pattern was sufficient to bring about learning the movement pattern resulting with better performance on a delayed retention test compared to a group that was passively guided through the movement pattern [81]. However, partial guidance performance was less accurate when compared to a group that practiced the task with no assistance at all [81]. The presence of extra partial guidance via force channels experienced by the Augmented PF group relative to the VF group accounted for approximately 11% of the total practice dose (32/295 reaches per session). Group differences with respect to the relative amount of partial guidance received via force channels may have contributed to performance advantage observed for the VF group associated with movement timing for the unilateral and bimanual VGR assessments.

### Limitations

The presence of motor and position matching impairments were also observed in the less-affected arm. Despite evidence of abnormality relative to normative values, unilateral VGR task scores and unilateral position matching accuracy metrics were still less-impaired relative to the stroke-affected side. We acknowledge that presence of somatosensory impairment in the less-affected arm would be a limitation for an intervention that relies on a reliable position sense signal from the less-affected arm. We observed that less-affected arm position matching ability (measured with the OAP assessment) was not related to magnitude of change in stroke-affected arm position sense (measured with APM). This finding suggests that the presence of less-affected upper limb impairment did not diminish or interfere with the intervention effect on position sense for the stroke-affected arm. It is worth emphasizing that the OAP normative data set (see Supplementary Materials) is different from the one used in the APM calculations for the stroke-affected hand (see [31]). The OAP normative data does not adjust for age, sex and handedness, so is likely more strict than the Kinarm standardized assessment models. APM and OAP assess accuracy and variability to the same target coordinates, permitting direct comparison of raw scores (also summarized in Table 1). For example, Absolute XY Error of matching performance for the stroke-affected hand with the APM task was almost double the magnitude of the matching performance of the less-affected hand measured with OAP. The net result is that less-affected upper limb somatosensory impairment is likely being overestimated. Future work could develop this OAP task further to establish ranges of severity and establish correlates with valid and reliable clinical measures of position matching performance.

## Conclusions

Our method of augmenting position sense feedback of the stroke-affected upper limb did result in improved spatiotemporal reach symmetry during training but did not provide additional benefit compared to uncoupled symmetrical reaching on our transfer assessments. Both training conditions transferred to improvement in upper limb motor impairment but not motor function, likely due to task-specific training effects. Importantly, both training conditions transferred to improved position matching ability by reducing APM variability. There was a trend for greater improvements in position sense after practicing with force-coupling for those with more severe baseline position sense impairment, highlighting potential individual differences in response. Replicating this study with a larger sample of individuals with clinically confirmed upper limb proprioceptive impairment in their stroke-affected arm would clarify whether force-coupling can effectively remediate proprioceptive deficits. Furthermore, inclusion of validated assessments that can accurately and reliably screen for position sense impairments in the less-affected upper limb could help guide group stratification or subgroup analyses to assess to what extent this intervention is beneficial to individuals whose position sense impairments present in both arms.

## Supplementary Information

Below is the link to the electronic supplementary material.

Supplementary Material 1.

Supplementary Material 2.

## Data Availability

All data will be available from the corresponding author upon reasonable request.
