# Correction: The impact of bimanual reach training with augmented position sense feedback on post-stroke upper limb sensory and motor impairment

**DOI:** 10.1186/s12984-025-01866-8

**Published:** 2026-02-09

**Authors:** Beverley C. Larssen, Ronan Denyer, Mahta Khoshnam Tehrani, Anjana Rajendran, Carlo Menon, Lara Boyd

**Affiliations:** 1 Graduate Program in Rehabilitation Sciences, University of British Columbia, Vancouver, BC Canada; 2 Graduate Program in Neuroscience, University of British Columbia, Vancouver, BC Canada; 3 Institute of Neuroscience, Université catholique de Louvain, Brussels, 1200 Belgium; 4 Biomedical and Mobile Health Technology Laboratory, Department of Health Sciences and Technology, ETH Zurich, Lengghalde 5, Zurich, 8008 Switzerland; 5 Menrva Research Group, Schools of Engineering Science and Mechatronic Systems Engineering, Simon Fraser University, Metro Vancouver, BC Canada; 6 Department of Physical Therapy, University of British Columbia, Vancouver, BC V6T 1Z4 Canada

**Correction: Larssen et al. Journal of NeuroEngineering and Rehabilitation (2025) 22:260**

10.1186/s12984-025-01764-z

Following publication of the original article [1], the following errors were reported. The original article has been corrected.

**Table:** 

Section and paragraph	Incorrect text	Correct text
Training task performance	Change “of” to “on” in the sentence “ Adapting training task difficulty base of…….(Fugl-Meyer scores: 18–64).”	Corrected sentence should be: Adapting training task difficulty based **on** maximum active reach distance and area achieved by the stroke-affected arm in the Kinarm workspace accommodated participants with a range of severe to mild upper limb motor impairments (Fugl-Meyer scores: 18–64).
Transfer to assessment of unilateral reaching	Delete “>” in i. > Unilateral VGR Task Score	i. Unilateral VGR Task Score
Figure 4 Caption	Change “task score” to “APM Task Score”.	The corrected sentence in the Figure caption should be: Individual subject scores are represented by individual points on the plot, where triangles represent individuals whose baseline **APM Task Score** was impaired relative to normative values (> 1.65).
Section heading	Colon missing in the section heading “Transfer to assessment of bilateral reaching Bimanual VGR Interlimb RMSE” after the word “Bimanual”.	Corrected section heading should be: “Transfer to assessment of bilateral reaching: Bimanual VGR Interlimb RMSE”
Section heading	Delete ‘.’ after the section heading “Transfer to clinical assessments of unilateral upper limb motor impairment and function.”	Transfer to clinical assessments of unilateral upper limb motor impairment and function
Incorrect reference link	F1 is incorrectly linked to a reference, delete 1 from F in the sentence. F1(1, 22) = 6.15, *p* = .02,$\:{\eta\:}_{p}^{2}$= 0.22.	Corrected sentence should read " *F*(1, 22) = 6.15, *p* = .02,$\:{\eta\:}_{p}^{2}$= .22”
Reference	Delete ‘.’ from the author name ‘Dexterit–E’ in the reference 31.	Cirrected reference should read as: 31. Kinarm, Dexterit-E 3.9 Addendum: Kinarm Standard Tests Summary [Internet]. Kingston; 2021. Available from: https://kinarm.com/download/kst-summary-analysis-version-3-9/
