# Supplementary material for: The impact of bimanual reach training with augmented position sense feedback on post-stroke upper limb somatosensory and motor impairment
Source: J Neuroeng Rehabil. 2025 Dec 9;22:260. doi: 10.1186/s12984-025-01764-z (PMC12690791; doi:10.1186/s12984-025-01764-z)
Supplement: Supplementary file 1 — Supplementary Material 1. [file 12984_2025_1764_MOESM1_ESM.pdf]

Task Name	Description	# of Target Positions (per arm)	# of Trials	Variation	Knowledge of results	Criteria for success	Example
Active Range of Motion (ROM) Assessment	Each arm is assessed separately. Participants attempt to reach all illuminated circular targets (2 cm radius) to determine range of motion (ROM) at the start of each session.	30	1 per arm	None.	Circles change color when touched.	None.<br><br>Distance reached during ROM assessment was used to inform selection of task difficulty (Easier → Harder) on subsequent custom reaching tasks based on visual aid below:<br>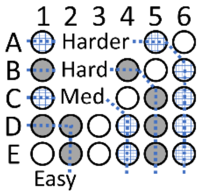 	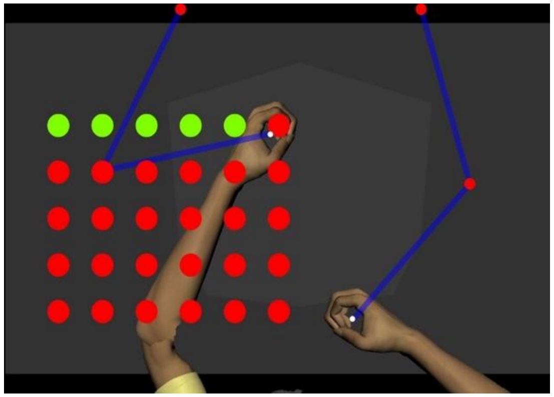<br><br><u>Instructions:</u> *This task shows you how far you can reach. The goal of this task is to reach to all of the green circle targets as quickly as you can.*<br>*When you reach each target they will change colour from green to red. First, they will appear on the (right or left depending on which side is unaffected) and then the (stroke-affected side).*<br>*When the targets appear on the right, use only your right arm and when the targets appear on the left, I want you to use only your left arm. You can begin reaching as soon as the targets appear on the screen.*
Implicit Sequence Learning Task	Mirrored stationary targets are presented on both sides of the screen. Participants perform planar reaches to targets as soon as they appear.	9	111	<u>Augmented PF group:</u><br>Proportional force feedback is transmitted to the less-affected arm to slow down or accelerate its movement to match the stroke-affected arm. Visual feedback of the position of the stroke-affected hand is turned off.	Targets change color when touched.	For both groups, the goal in this task is to move both arms together as quickly and as accurately as possible to reach the targets.	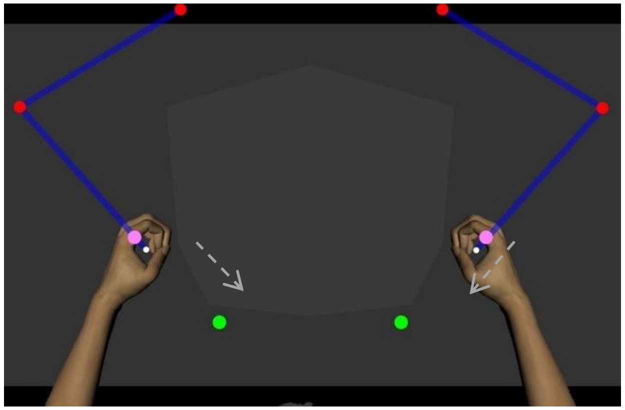<br><br><u>Instructions</u>: *For this task, your hand position is represented by a white dot. You will see the white dot for both hands. Move both hands to the green targets to begin. For this task, you will see the same targets on both sides of the screen.*<br>*The goal of the task is to move your arms together as fast and accurately as possible from one pair of green targets to the following pair of green targets. To the best of your ability try to move your arms at the same time and speed to reach the targets projected on the screen.*<br>*You can start as soon as the first set of green targets appear.*

Mirror Extension Task	Mirrored stationary targets are presented on both sides of the screen. Participants perform planar reaches (extension, away from midline) to targets as soon as they appear.	8	32	<u>Static task difficulty:</u> Distance reached scaled based on ROM task performance. Possible target distances (10 cm – 25 cm).<br><br><u>Dynamic task difficulty:</u> Radius of targets starts at 3 cm. For every 3 consecutive successful target reaches, radius decreases in 1 cm steps to a minimum of 1 cm. For every 3 consecutive missed reaches (target not reached in allotted trial time), target radius increased in steps of 1 cm to maximum of 5 cm.<br><br><u>Augmented PF group:</u> Proportional force feedback is transmitted to the less-affected arm to slow down or accelerate its movement to match the stroke-affected arm. On 50% of the trials, a force channel is implemented between start and end points for each arm to prevent deviation from a straight path. Visual feedback of the position of the stroke-affected hand is turned off.	Picture of coffee cups appear behind green circle targets. If targets are reached successfully, “Enjoy Your Coffee” text appears in the place of the coffee cups. If targets are not reached in the allotted trial time (15 seconds), an image of spilled coffee cups appear in the place of the coffee cups.	The goal in this task is to extend both arms simultaneously to targets away from the participant’s midline.	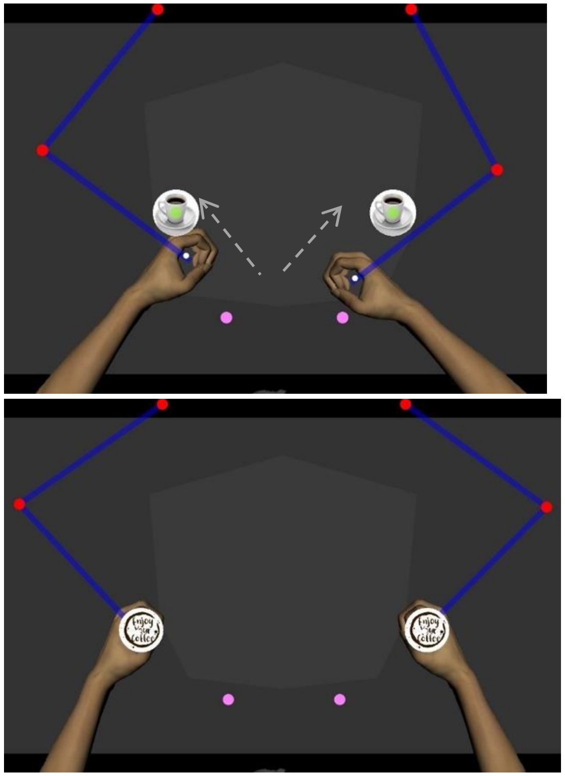<br><u>Instructions</u>: *For this task, your hand position is represented by a white dot. You will see the white dot for both hands.*<br>*For this task, you will see the same targets on both sides of the screen. The goal of the task is to move your arms together as fast and accurately as possible from the first set of green targets in the center to the target coffee cups which will appear at the periphery. The green targets within the coffee cups will get smaller as time goes on so it is important to not only reach to the coffee cups but to accurately reach to the green targets within the coffee cups. Once both hands are inside the green targets, a new pair of green targets will reappear at the center of the screen and the next trial will begin.*<br>*To the best of your ability try to move your arms at the same time and speed to reach the targets projected on the screen. You can start as soon as the first set of green targets appear.*

Mirror Flexion Task	Mirrored stationary targets are presented on both sides of the screen. Participants perform planar reaches (flexion, towards midline) to targets as soon as they appear.	8	32	<u>Static task difficulty:</u> Distance reached scales with ROM task performance. Same parameters as Mirror Extension Task.<br><br><u>Dynamic task difficulty:</u> As per criteria and parameters described for Mirror Extension Task.<br><br><u>Augmented PF group:</u> Proportional force feedback is transmitted to the less-affected arm to slow down or accelerate its movement to match the stroke-affected arm. On 50% of the trials, a force channel is also implemented between start and end points for each arm to prevent deviation from a straight path. Visual feedback from position of the stroke-affected hand is turned off.	Picture of pens appear behind target. If targets are reached successfully, "You Got This" text appears in the place of the pens. If targets are not reached in the allotted trial time (15 seconds), an image of "Oops" text appear in the place of the pens.	The goal in this task is to flex both arms simultaneously towards targets located near the participant's midline.	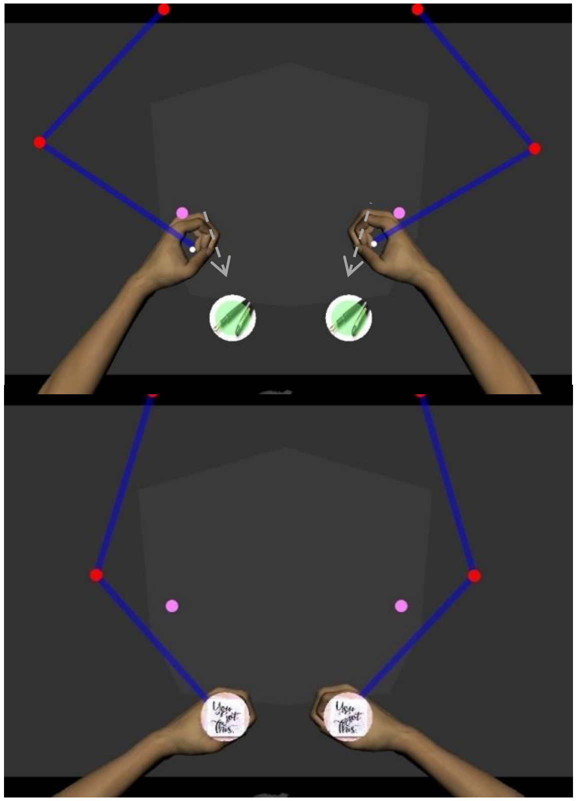<br><u>Instructions</u>: *For this task, your hand position is represented by a white dot. You will see the white dot for both hands. For this task, you will see the same targets on both sides of the screen. The goal of the task is to move your arms together as fast and accurately as possible from the first set of green targets to the image of pens in the center of the screen. The center of each image is marked with a green target which will get smaller as time goes on.*<br>*Once both hands are inside the green targets, a new pair of green targets will reappear at the periphery and the next trial will begin. To the best of your ability try to move your arms at the same time and speed to reach the targets projected on the screen.*<br>*You can start as soon as the first set of green targets appear.*

Intercepting Moving Targets - 1	Mirrored pairs of moving targets descend from top of screen. Participants place both arms at their corresponding start points. Two circles ("balls"; 1.5 cm radius) fall in a straight line from top of the screen at the same speed and relative position. Participants move both hands simultaneously to catch the falling circles. The hands must maintain the same distance between them throughout the task. For both groups, a force channel is also implemented to only permit mediolateral movement (along the x-axis) and prevent vertical movements of the hands. See footnote for rationale for force channel implementation.[1]	10	40	<u>Dynamic task difficulty:</u> Achieved via manipulation of moving target speed, and length of paddles. Starting target speed: 5 cm/s. Starting Paddle width: 7 cm.<br><br>For every 3 consecutive successfully intercepted target pairs, the speed of the descending targets increases by steps of 3 cm/s up to a maximum of 20 cm/s. Then paddle length decreases in steps of 1 cm until a minimum of 2 cm. and the width of the virtual paddles becomes smaller.<br><br>If the first 3 targets are missed, the speed of the moving targets decreases first to 2 cm/s, then paddle length increases in steps of 1 cm to a maximum of 10 cm, for subsequent trios of trial failures.<br><br><u>Augmented PF group:</u> The less-affected arm is force-coupled to the stroke-affected arm to maintain a fixed distance between hands about vertical axis.	When hands are correctly positioned and successfully intercept both circles, the circle will appear to bounce back away from the hands.<br><br>When hands are positioned incorrectly, both circles will fall to bottom of the screen.<br><br>All participants are provided with a score out of 40 at the end of the task to inform them of the total number of successful interceptions.	The goal is to move both hands simultaneously in the mediolateral direction to intercept the falling circles. The hands should maintain the same distance throughout the task and the same y-coordinates at the time of target interception (y-coordinates are constrained by the presence of a force channel for both groups).	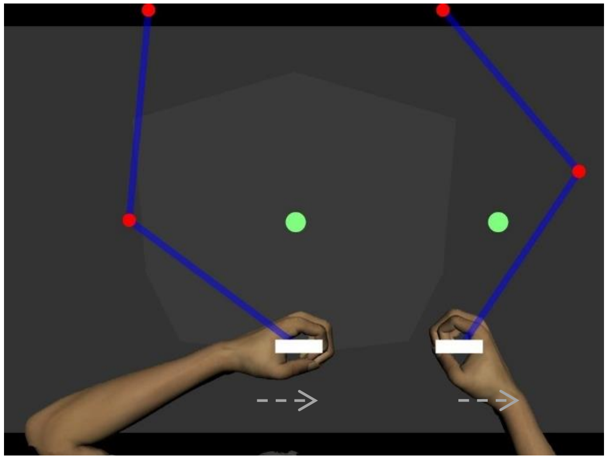<br><br><u>Instructions</u>: *For this task, your hand position is represented by a white rectangular paddle.*<br><br>***Augmented PF group only****: [You will only see the white paddle for your [unaffected] arm. Move both hands to the green rectangular targets to begin.*<br>*Once you've reached to the green rectangles, your arms will be linked together so that when you move your [stroke-affected] arm your [unaffected] arm will move with it to perform the mirror-matched movement.]*<br><br>*The goal of the task is to move your arms together as fast and accurately to bounce balls away as they fall from the top of the workspace towards you. Two balls will fall at once, one on each side of the workspace. Your hands must be in line with one another and must both be under each ball in order to accurately bounce the balls away.*<br>*The relationship between your arms has changed – you must now move your arms like windshield wipers*<br><br>*(experimenter gestures motion using own arms).*<br><br>*To the best of your ability try to move your arms at the same time and speed to hit the moving targets projected on the screen.*<br><br>***Augmented PF group****: [If your arms are not moving together and at the same speed, you will feel a force on your [unaffected] arm. You will not see where your impaired hand is so use the force felt in your unaffected hand as your guide to get both hands to the moving targets.]*<br><br>*You can start as soon as the first set of green targets appear.*

				Visual feedback of the position of the stroke-affected hand is turned off.			
Intercepting moving targets - 2	Mirrored pairs of moving targets approach from the side of the screen. Participants place both arms at their corresponding start points. Two circles (“balls”; 1.5 cm radius) will move horizontally, one from the left and one from the right side of the screen, toward its vertical center line at the same speed and relative position. Participants move both hands simultaneously to catch the moving circles. The hands must maintain the same distance between them throughout the task. For both groups, a force channel is also implemented to only permit movement along the y-axis and prevent horizontal movements of the hands. See footnote for rationale for force channel implementation.[1]	10	40	<u>Dynamic Task Difficulty:</u> As per criteria and parameters described for Intercepting moving targets – 1.<br><br><u>Augmented PF group:</u> the less-affected arm is force-coupled to the stroke-affected arm to maintain mirrored position of the stroke-affected hand at a fixed distance from the horizonal center. Visual feedback of the position of the stroke-affected hand is turned off.	When hands are correctly positioned and successfully intercept both circles, the circle will appear to bounce back away from the hands.<br><br>When hands are positioned incorrectly, both circles will fall to bottom of the screen.<br><br>All participants are provided with a score out of 40 at the end of the task to inform them of the total number of successful interceptions.	The goal in this task is to flex/extend two arms simultaneously in the vertical direction to intercept the moving circles. Depending on the location of moving circles, the hands move either towards or away from the body. The hands must achieve symmetrical x- and y-coordinates at the time of target interception (x-coordinates are constrained by the presence of a force channel for both groups).	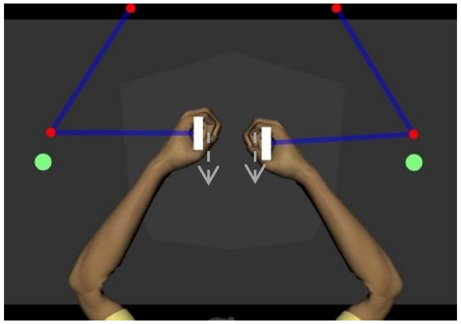<br><u>Instructions</u>: *For this task, your hand position is represented by a white rectangular paddle. You will see the white paddles for both hands. For this task, you will see the same targets on both sides of the screen. This task is similar to the previous task, except the balls are coming from the sides of the screen as opposed to from the top of the screen. You will now be required to move your arms up and down on the screen.*<br><br>***Augmented PF group only****: [For this task, your hand position is represented by a white rectangular paddle. You will only see the white paddle for your [unaffected] arm. Move both hands to the green rectangular targets to begin. Once you've reached to the green rectangles, your arms will be linked together so that when you move your [stroke-affected] arm your [unaffected] arm will move with it to perform the mirror-matched movement.]*<br><br>*The goal of the task is to move your arms together as fast and accurately as possible to bounce balls away as they come from the side of the workspace towards you.*<br>*Two balls will fall at once, one on each side of the workspace. Your hands must be in line with one another and must both be in-line with each ball in order to accurately bounce the balls away.*<br>*To the best of your ability try to move your arms at the same time and speed to hit the moving targets projected on the screen.*<br><br>***Augmented PF group****: [If your arms are not moving together and at the same speed, you will feel a force on your [unaffected] arm. You will not see where our impaired hand is so use the force felt in your unaffected hand as your guide to get both hands to the moving targets.]*<br><br>*You can start as soon as the first set of green targets appear.*

Intercepting moving targets - 3	Mirrored pairs moving targets descend from top of screen. Participants place both arms at their corresponding start points. Two circles (“balls”) fall in a straight line from top of the screen at the same speed and relative position. Participants move both hands simultaneously to catch the falling circles. The hands should maintain the same distance with respect to the vertical center line throughout the task. For both groups, a force channel is also implemented to only permit mediolateral movement (along the x-axis) and prevent vertical movements of the hands. See footnote for rationale for force channel implementation.[1]	10	40	<u>Dynamic Task Difficulty:</u> As per criteria and parameters described for Intercepting moving targets – 1.<br><br><u>Augmented PF group:</u> The less-affected arm is force-coupled to the stroke-affected arm to maintain mirrored position of the stroke-affected hand at a fixed distance from the vertical center. Visual feedback of the position of the stroke-affected hand is turned off.	When hands are correctly positioned and successfully intercept both circles, the circle will appear to bounce back away from the hands.<br><br>When hands are positioned incorrectly, both circles will fall to bottom of the screen.<br><br>All participants are provided with a score out of 40 at the end of the task to inform them of the total number of successful interceptions.	The goal in this task is to move two hands simultaneously in the horizontal (medial-lateral) direction to intercept the falling circles. Depending on the location of falling circles, the hands move either towards or away from each other. The hands must achieve symmetrical x- and y-coordinates at the time of target interception (y-coordinates are constrained by the presence of a force channel for both groups).	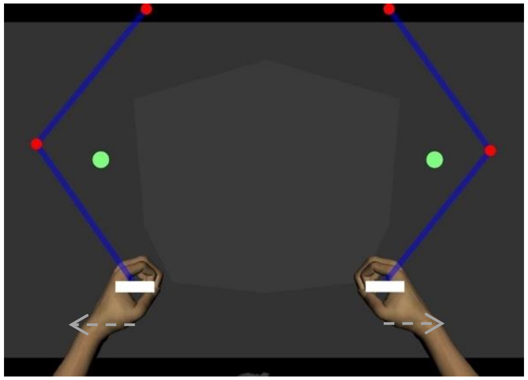<br><u>Instructions</u>: *For this task, your hand position is represented by a white rectangular paddle. Move both hands to the green rectangular targets to begin.*<br><br>***Augmented PF group only****: [You will only see the white paddle for your [unaffected] arm. Once you’ve reached to the green rectangles, your arms will be linked together so that when you move your [stroke-affected] arm your [unaffected] arm will move with it to perform the mirror-matched movement.]*<br><br>*The goal of the task is to move your arms together as fast and accurately to bounce balls away as they fall from the top of the workspace towards you. Two balls will fall at once, one on each side of the workspace. Your hands must be in line with one another and must both be under each ball in order to accurately bounce the balls away.*<br>*To the best of your ability try to move your arms at the same time and speed to hit the moving targets projected on the screen.*<br><br>***Augmented PF group only****: [If your arms are not moving together and at the same speed, you will feel a force on your [unaffected] arm. You will not see where our impaired hand is, so use the force felt in your unaffected hand as your guide to get both hands to the moving targets.]*<br><br>*You can start as soon as the first set of green targets appear.*

[1] The decision to implement a force channel for both groups in the intercepting targets tasks was to reduce the task difficulty by limiting the active degrees of freedom controlled by the participants to one movement axis. This was informed during piloting of the tasks, where we observed that it was too difficult for participants with stroke to accurately intercept moving targets when free control of both x- and y-coordinates was permitted for both hands (equivalent to the VF group task conditions without a force channel present). Simplifying the task ensured more opportunities for participants to experience task success.
