# Supplementary material for: The impact of bimanual reach training with augmented position sense feedback on post-stroke upper limb somatosensory and motor impairment
Source: J Neuroeng Rehabil. 2025 Dec 9;22:260. doi: 10.1186/s12984-025-01764-z (PMC12690791; doi:10.1186/s12984-025-01764-z)
Supplement: Supplementary file 2 — Supplementary Material 2. [file 12984_2025_1764_MOESM2_ESM.pdf]

Supplementary Materials

One arm position matching assessment

A sample of 35 control participants ages 18 – 76 years old without a history of stroke completed the custom One Arm Position matching assessment (OAP). The Kinam Arm Position Matching (APM) standardized assessment was also completed to confirm normal position matching abilities. Both assessments were completed with both hands. These data were collected as baseline measures from another study approved by the University of British Columbia Clinical Research Ethics Board (Study number: H21-03625). Written informed consent was obtained from all participants for inclusion of these data in secondary analyses. Based on APM Task Scores, accuracy z-scores, and variability z-scores, all control participants had normal position matching performance relative to normative models (Task Scores < 1.96 and parameter z-scores < 1.65 for both hands).

Mean OAP accuracy (mean Euclidean distance of handle position relative to target location) and variability (root sum of squares of the standard deviation of accuracy measures, OAP VarXY = $\sqrt{SDx^2+SDy^2}$) were calculated for right and left hands separately and then pooled across hands. The pooled average and standard deviation that is inclusive of right and left scores for each parameter was used as the reference for calculating z-score for the stroke participant data. Data from both hands were pooled to include equal samples for dominant and non-dominant hand performance. Parameter scores and participant demographics are summarized in Supplementary Table 2.

Since the same target locations were sampled for OAP and APM tasks, comparisons could be made between assessments for accuracy and variability measures. Participants had better accuracy scores (lower error) in the OAP matching task compared to APM for both the right ($t(34) = -7.46$, $p < 0.001$, $d = 1.60$) and left hand ($t(34) = -5.85$, $p < 0.001$, $d = 1.62$). Variability measures did not differ between assessments for either hand (Right: $t(34) = -1.52$, $p = 0.14$, $d = 0.81$; Left: $t(34) = 0.27$, $p = 0.79$, $d = 0.65$).

Supplementary Table 2. One arm position matching normative data set. Participant demographics and Kinarm assessment scores.

	Controls<br>n = 35		
Demographics			
Sex (Females/Males)	24/11		
Median Age, years (*IQR)*	39 (22, 56)		
Handedness (Right/Left)	33/2		
Kinarm APM Assessment Parameters	Right Hand<br>*n= 35 samples*	Left Hand<br>*n= 35 samples*	Both Hands (Pooled)
Mean APM Task Score (SD)	0.48 (0.40)	0.34 (0.42)	-
APM Accuracy:<br>Mean AbsXY cm (SD)	4.08 (1.50)	3.57 (1.58)	-
Mean AbsXY Z-Score (SD)	-0.39 (0.94)	-1.03 (1.33)	-
APM Variability:<br>Mean VarXY cm (SD)	2.45 (0.58)	2.12 (0.55)	-
Mean VarXY Z-Score (SD)	-0.62 (0.85)	-1.39 (1.10)	-
Kinarm OAP Assessment Parameters			
Mean OAP Accuracy, AbsXY cm (SD)	2.06 (0.50)	1.97 (0.42)	2.02 (0.46)
Mean OAP Variability, VarXY cm (SD)	2.25 (0.57)	2.15 (0.48)	2.20 (0.52)

APM = Arm Position Matching test, OAP = One Arm Position matching test, SD = standard deviation, IQR = inter-quartile range.

Active range of motion (ROM) assessment

See figure below for ROM assessment data. Panel A illustrates the maximum reach distances (centimeters, cm) and areas of target coverage (cm^2) required for each task difficulty level for the custom Mirror Extension task. Each data point represents the location of a target, plotted as a function of its target coordinates in cm. Reach distances achieved during the ROM task informed the selection of the corresponding level of task difficulty. Panel B illustrates the progression of task difficulty levels for each participant based on ROM (y-axis) across the 11 training sessions (3-13, x-axis). The group (Aug. PF = Augmented Position Sense Feedback; VF = Visual Feedback Control), participant number, and the participant's average baseline Fugl-Meyer score (/66) is provided for each participant. The different task difficulty levels permitted participation for a range of upper limb motor impairment severities. Task difficulty progressions were only required for 5 participants (B23, B26, B25, B27, B33).

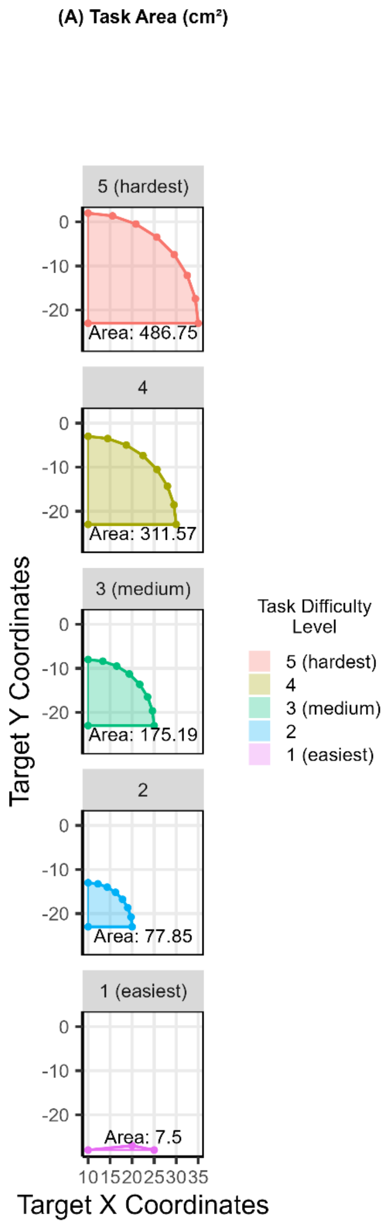

(A) Task Area (cm²)
5 (hardest)
Area: 486.75
4
Area: 311.57
3 (medium)
Area: 175.19
2
Area: 77.85
1 (easiest)
Area: 7.5
Target Y Coordinates
Target X Coordinates
Task Difficulty Level
5 (hardest)
4
3 (medium)
2
1 (easiest)

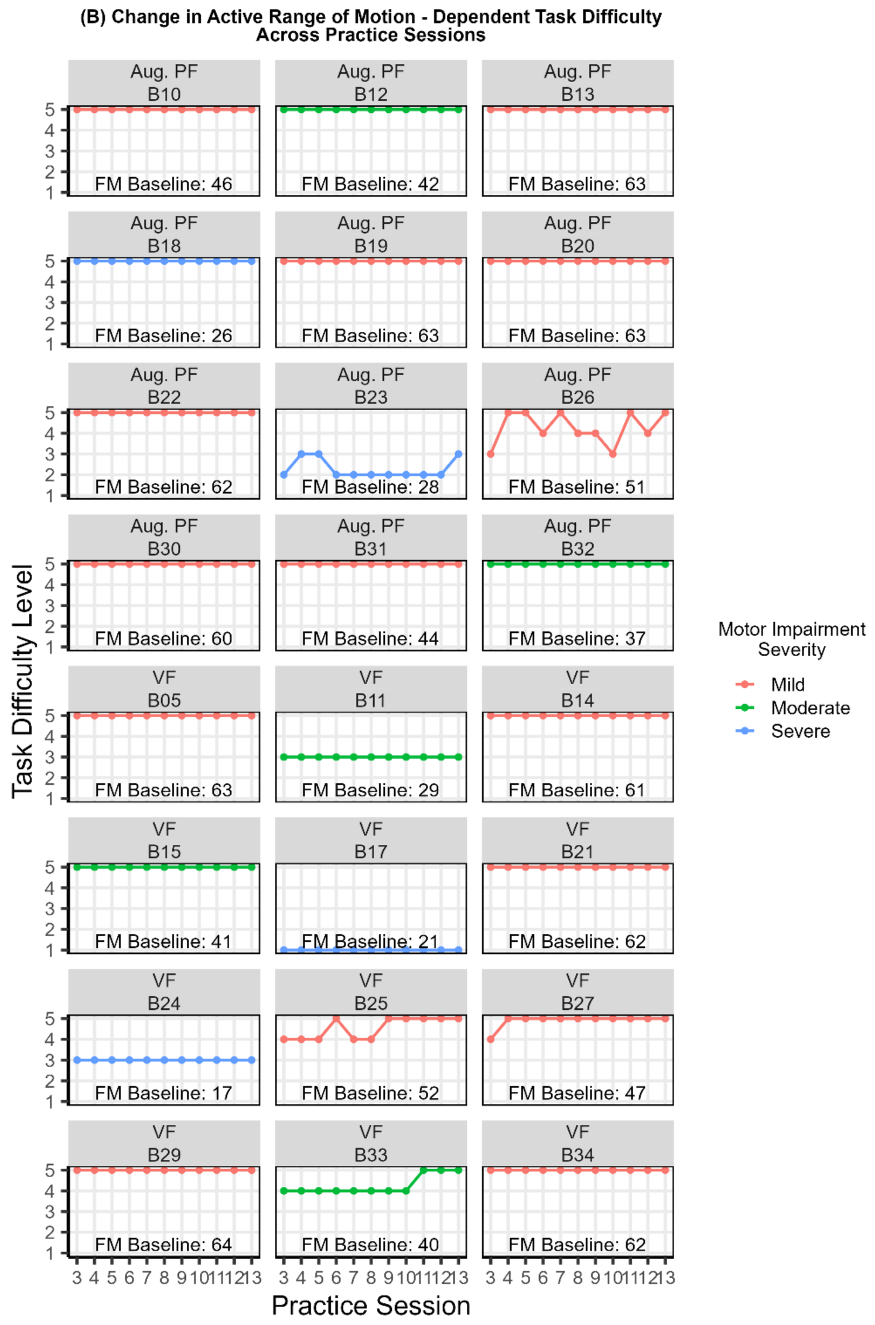

(B) Change in Active Range of Motion - Dependent Task Difficulty Across Practice Sessions
Aug. PF B10 FM Baseline: 46
Aug. PF B12 FM Baseline: 42
Aug. PF B13 FM Baseline: 63
Aug. PF B18 FM Baseline: 26
Aug. PF B19 FM Baseline: 63
Aug. PF B20 FM Baseline: 63
Aug. PF B22 FM Baseline: 62
Aug. PF B23 FM Baseline: 28
Aug. PF B26 FM Baseline: 51
Aug. PF B30 FM Baseline: 60
Aug. PF B31 FM Baseline: 44
Aug. PF B32 FM Baseline: 37
VF B05 FM Baseline: 63
VF B11 FM Baseline: 29
VF B14 FM Baseline: 61
VF B15 FM Baseline: 41
VF B17 FM Baseline: 21
VF B21 FM Baseline: 62
VF B24 FM Baseline: 17
VF B25 FM Baseline: 52
VF B27 FM Baseline: 47
VF B29 FM Baseline: 64
VF B33 FM Baseline: 40
VF B34 FM Baseline: 62
Task Difficulty Level
Practice Session
Motor Impairment Severity
Mild
Moderate
Severe
